# Supplementary material for: Markers of pubertal timing and leisure-time physical activity from ages 36 to 68 years: findings from a British birth cohort
Source: BMJ Open. 2017 Nov 1;7(10):e017407. doi: 10.1136/bmjopen-2017-017407 (PMC5695476; doi:10.1136/bmjopen-2017-017407)
Supplement: Supplementary table 1 [file bmjopen-2017-017407supp001.pdf]

Supplementary table 1 Associations between Pubertal Status at Age 15 Years and Leisure-Time Physical Activity (LTPA) at each Adult Age in Men from the MRC NSHD after further adjustment for adult physical health, 1946-2014.

	N (%) LTPA*	Odds ratios (95% confidence intervals) of LTPA at least once per month at each adult age versus no LTPA	
		Model A	Model B
LTPA age 36 years			
fully mature (n=316)	230 (72.8)	1.00	1.00
advanced stage puberty (n=395)	258 (65.3)	0.71 (0.51, 0.98)	0.69 (0.50, 0.96)
early stage puberty (n=455)	308 (67.7)	0.78 (0.57, 1.08)	0.76 (0.55, 1.05)
prepubescent (n=131)	97 (74.1)	1.12 (0.70, 1.78)	1.09 (0.69, 1.75)
test of association		*P*=0.07	*P*=0.06
LTPA age 43 years			
fully mature (n=293)	169 (57.7)	1.00	1.00
advanced stage puberty (n=363)	176 (48.5)	0.69 (0.50, 0.95)	0.68 (0.49, 0.94)
early stage puberty (n=408)	201 (49.3)	0.74 (0.54, 1.00)	0.71 (0.52, 0.98)
prepubescent (n=119)	58 (48.7)	0.78 (0.50, 1.21)	0.75 (0.48, 1.18)
test of association		*P*=0.1	*P*=0.1
LTPA age 53 years			
fully mature (n=258)	135 (52.3)	1.00	1.00
advanced stage puberty (n=314)	164 (52.2)	1.03 (0.73, 1.44)	1.01 (0.72, 1.42)
early stage puberty (n=367)	191 (52.0)	1.03 (0.74, 1.42)	0.98 (0.70, 1.36)
prepubescent (n=106)	54 (51.0)	1.05 (0.66, 1.67)	1.04 (0.65, 1.66)
test of association		*P*>0.9	*P*>0.9
LTPA age 60-64 years			
fully mature (n=195)	66 (33.9)	1.00	1.00
advanced stage puberty (n=235)	85 (36.2)	1.15 (0.76, 1.72)	1.13 (0.75, 1.71)
early stage puberty (n=272)	85 (31.3)	0.92 (0.61, 1.37)	0.87 (0.58, 1.31)
prepubescent (n=82)	23 (28.1)	0.84 (0.47, 1.51)	0.84 (0.46, 1.50)
test of association		*P*=0.6	*P*=0.5
LTPA age 68 years			
fully mature (n=203)	80 (39.4)	1.00	1.00
advanced stage puberty (n=253)	98 (38.7)	0.97 (0.66, 1.42)	0.96 (0.66, 1.42)
early stage puberty (n=289)	114 (39.5)	0.98 (0.68, 1.43)	0.97 (0.67, 1.41)
prepubescent (n=84)	31 (36.9)	0.94 (0.55, 1.60)	0.94 (0.55, 1.60)
test of association		*P*>0.9	*P*>0.9

Analytic samples consist of those with maximum data at each age. Model A: adjusted for birth weight, birth order, childhood illness and father's occupational class. Model B: further adjusted for adult physical health. P-values from test of difference between pubertal groups.
