# Supplementary material for: Markers of pubertal timing and leisure-time physical activity from ages 36 to 68 years: findings from a British birth cohort
Source: BMJ Open. 2017 Nov 1;7(10):e017407. doi: 10.1136/bmjopen-2017-017407 (PMC5695476; doi:10.1136/bmjopen-2017-017407)
Supplement: Supplementary table 2 [file bmjopen-2017-017407supp002.pdf]

Supplementary table 2 Associations between Age at Menarche and Leisure-Time Physical Activity (LTPA) at each Adult Age in Women from the MRC NSHD after further adjustment for adult physical health, 1946-2014.

	N (%) LTPA*	Odds ratios (95% confidence intervals) of LTPA at least once per month at each adult age versus no LTPA	
		Model A	Model B
LTPA age 36 years			
≤ 11 (n=210)	115 (54.8)	1.00	1.00
12 (n=359)	200 (55.7)	1.07 (0.75, 1.52)	1.03 (0.72, 1.47)
13 (n=457)	270 (59.1)	1.22 (0.87, 1.72)	1.18 (0.84, 1.67)
≥ 14 (n=235)	131 (55.7)	1.08 (0.73, 1.58)	1.00 (0.67, 1.47)
test of association		*P*=0.6	*P*=0.6
LTPA age 43 years			
≤ 11 (n=190)	74 (39.0)	1.00	1.00
12 (n=333)	143 (42.9)	1.25 (0.85, 1.79)	1.22 (0.84, 1.77)
13 (n=407)	173 (42.5)	1.22 (0.85, 1.76)	1.19 (0.83, 1.71)
≥ 14 (n=219)	99 (45.2)	1.36 (0.91, 2.04)	1.30 (0.86, 1.97)
test of association		*P*=0.5	*P*=0.6
LTPA age 53 years			
≤ 11 (n=184)	92 (50.0)	1.00	1.00
12 (n=305)	148 (48.5)	1.01 (0.69, 1.47)	0.99 (0.68, 1.45)
13 (n=377)	170 (45.1)	0.89 (0.61, 1.28)	0.87 (0.60, 1.26)
≥ 14 (n=213)	109 (51.2)	1.13 (0.75, 1.70)	1.08 (0.71, 1.63)
test of association		*P*=0.6	*P*=0.6
LTPA age 60-64 years			
≤ 11 (n=133)	44 (33.1)	1.00	1.00
12 (n=248)	95 (38.3)	1.50 (0.94, 2.39)	1.44 (0.90, 2.30)
13 (n=290)	112 (38.6)	1.56 (0.99, 2.45)	1.54 (0.97, 2.42)
≥ 14 (n=157)	56 (35.7)	1.26 (0.75, 2.09)	1.15 (0.69, 1.93)
test of association		*P*=0.2	*P*=0.2
LTPA age 68 years			
≤ 11 (n=142)	55 (38.7)	1.00	1.00
12 (n=250)	96 (38.4)	1.06 (0.68, 1.63)	1.02 (0.66, 1.57)
13 (n=323)	133 (41.2)	1.15 (0.76, 1.74)	1.13 (0.75, 1.72)
≥ 14 (n=164)	66 (40.2)	1.11 (0.69, 1.78)	1.03 (0.64, 1.66)
test of association		*P*>0.9	*P*=0.9

Analytic samples consist of those with maximum data at each age. Model A: adjusted for birth weight, birth order, childhood illness and father's occupational class. Model B: further adjusted for adult physical health. P-values from test of difference between pubertal groups.
