# Supplementary material for: Markers of pubertal timing and leisure-time physical activity from ages 36 to 68 years: findings from a British birth cohort
Source: BMJ Open. 2017 Nov 1;7(10):e017407. doi: 10.1136/bmjopen-2017-017407 (PMC5695476; doi:10.1136/bmjopen-2017-017407)
Supplement: Author's manuscript [file bmjopen-2017-017407.draft_revisions.pdf]

BMJ Open is committed to open peer review. As part of this commitment we make the peer review history of every article we publish publicly available.

When an article is published we post the peer reviewers' comments and the authors' responses online. We also post the versions of the paper that were used during peer review. These are the versions that the peer review comments apply to.

The versions of the paper that follow are the versions that were submitted during the peer review process. They are not the versions of record or the final published versions. They should not be cited or distributed as the published version of this manuscript.

BMJ Open is an open access journal and the full, final, typeset and author-corrected version of record of the manuscript is available on our site with no access controls, subscription charges or pay-per-view fees (http://bmjopen.bmj.com).

If you have any questions on BMJ Open's open peer review process please email editorial.bmjopen@bmj.com

BMJ Open

Is age at puberty associated with leisure-time physical activity across adulthood? Findings from a British birth cohort

Journal:	*BMJ Open*
Manuscript ID	bmjopen-2017-017407
Article Type:	Research
Date Submitted by the Author:	21-Apr-2017

Complete List of Authors:	Elhakeem, Ahmed; University of Bristol School of Clinical Science,<br>Cooper, Rachel; UCL, MRC Unit for Lifelong Health and Ageing at UCL<br>Bann, David; University College London, UCL Institute of Education<br>Kuh, Diana; MRC Unit for Lifelong Health and Ageing at UCL,<br>Hardy, Rebecca; University College London, MRC Unit for Lifelong Health and Ageing

**Primary Subject Heading**:	Epidemiology
Secondary Subject Heading:	Public health
Keywords:	Life course, leisure-time, physical activity, pubertal timing

SCHOLARONE™ Manuscripts

For peer review only

Is age at puberty associated with leisure-time physical activity across adulthood? Findings from a British birth cohort

Ahmed Elhakeem, PhD[1,2]*, Rachel Cooper, PhD[1], David Bann, PhD[3], Diana Kuh, PhD[1], Rebecca Hardy, PhD[1]

[1]MRC Unit for Lifelong Health and Ageing at UCL, London, UK

[2]Musculoskeletal Research Unit, School of Clinical Sciences, University of Bristol, Bristol, UK

[3] Centre for Longitudinal Studies, UCL Institute of Education, London, UK

*Correspondence: Ahmed Elhakeem, Musculoskeletal Research Unit, School of Clinical Sciences, University of Bristol, Level 1 Learning and Research Building, Southmead hospital, Bristol BS10 5NB, UK, Email: a.elhakeem@bristol.ac.uk, Tel: 0044(0)1174147864

Abstract = 286 words, Text = 2926 words, References = 35, Tables = 3, Figures = 2

For peer review only

ABSTRACT

Objectives

The aim of this study was to examine the associations between age at puberty and leisure-time physical activity (LTPA) across adulthood in British men and women.

Study design

Data were from the MRC National Survey of Health and Development. Boys' pubertal status and girls' age at menarche were ascertained by physicians at age 14-15. Boys were grouped as prepubescent, early puberty, advanced puberty or fully mature. Girls were grouped as reaching menarche ≤11 years, 12 years, 13 years or ≥14 years. LTPA was reported at ages 36, 43, 53, 60-64 and 68 and classified as active or inactive at each age. Associations were examined using standard and mixed-effects logistic regression models.

Results

A total of 1499 men and 1409 women had at least one measure of adulthood LTPA and complete data on age at puberty and selected covariates. Weak evidence was found of an association between earlier maturation in boys and higher likelihood of LTPA at younger but not older adult ages (*P*=0.05 for pubertal status-by-age at LTPA interaction). For example, fully-adjusted odds ratios (OR) of LTPA (versus no LTPA) at ages 36 and 68 years respectively for advanced puberty versus fully mature were 0.69 (95% confidence intervals: 0.50, 0.96) and 1.03 (0.72, 1.47). Age at menarche was not associated with LTPA across adulthood (*P*≥0.7 for age at menarche-by-age at LTPA interaction). For example, OR of LTPA across adulthood (between 36 and 68 years) was 1.21 (0.91, 1.61) for reaching menarche at 13 versus ≤11 years.

Conclusions

For peer review only - http://bmjopen.bmj.com/site/about/guidelines.xhtml

In a nationally representative study, there was little evidence to suggest that timing of puberty is an important correlate of LTPA across adulthood. Maturity-related variations in adolescents' PA may represent a transitory effect which loses importance over time.

KEY WORDS

Life course; leisure-time; physical activity; pubertal timing.

STRENGTHS AND LIMITATIONS OF THIS STUDY

- Prospective assessment of pubertal timing in both males and females
- Leisure-time physical activity repeatedly measured throughout adulthood.
- Limited by fewer numbers of participants at the extremes of pubertal age.
- Birth cohort effects and contextual differences in what leisure-time physical activity signifies in adolescence and adulthood may contribute to findings.

For peer review only

For peer review only - http://bmjopen.bmj.com/site/about/guidelines.xhtml

BACKGROUND

Leisure-time physical activity (LTPA) promotes health and therefore it is important to identify groups that might benefit from support to participate in LTPA. Adolescence is a time when many health behaviours, such as LTPA habits are initiated (1, 2) and which can track into adulthood (3). Systematic reviews of cross-sectional studies suggest that early maturing girls participate less, and early maturing boys more, in sports activities in adolescence than those who reach puberty around the average age (4, 5), and it is thought that discomfort caused by menarche in early maturing girls and lower perceived competence at sports by later maturing boys may contribute to these findings (4-8). However, it is unclear if these maturity-related differences in adolescents LTPA are observed in adults.

Both earlier and later timing of puberty have also been associated with adverse health outcomes in later life which could influence participation in LTPA in adult life. For example, findings from UK Biobank showed both earlier and later ages at puberty were associated with a range of chronic disease outcomes, including heart disease and psychiatric disorders, in both men and women (9). In the MRC National Survey of Health and Development (NSHD), earlier puberty was associated in midlife with greater body mass index (10), higher levels of triglycerides in women (11) and higher blood pressure (10), and greater android fat mass in men (12), whereas later puberty was associated with less bone mineral density (13).

To our knowledge, only three studies have examined associations of age at puberty with LTPA in adults. Perceived relative pubertal status reported by 12-19-year-old Norwegians was not related to LTPA over thirteen years of follow-up in one study (14), and in the 1958 National Child Development Study (NCDS), age at puberty was unrelated to midlife LTPA (15). However, among 166 40-year old Belgian men (16), later age at peak height velocity (APHV), a marker of later pubertal onset, correlated with higher participation in sports. It is

For peer review only - http://bmjopen.bmj.com/site/about/guidelines.xhtml

possible that associations between pubertal timing and LTPA weaken with age, as the pathways on which puberty acts become less important influences on LTPA, or possibly due to health conditions associated with early or late puberty being associated with declines or improvement in health and LTPA. Therefore, detailed studies with repeat measures of LTPA across adult life are needed, including those that examine associations in both male and female samples. This study examines associations of age at puberty in both sexes with LTPA across adulthood, from ages 36 to 68 years, and whether any associations found change with age at assessment of LTPA.

METHODS

Participants

The NSHD is a nationally representative sample of 5,362 British singleton births during one week in March 1946 and followed up regularly across childhood and adulthood (17, 18). At ages 36 (1982), 43 (1989) and 53 (1999) trained nurses interviewed and assessed the study participants in their own homes. At age 60-64 (2006-2010) they attended one of 6 clinical research facilities or received a home visit, and at age 68 (2014) they completed a postal questionnaire followed by a home visit.

Of those successfully contacted at ages 36 (n=3322), 43 (n=3262), 53 (n=3035), 60-64 (n=2661) and 68 years (n=2453), 99.6%, 100%, 98.4%, 82.2% and 99.1% respectively provided information on LTPA (3766 participants had at least one measure of LTPA). The participating samples at ages 43, 53 and 60-64 have been found to be broadly representative of similar aged members of the general UK population (19-21). At the most recent round of data collection at age 68, of the 2816 people in the target sample living in England, Scotland and Wales, 2370 (84.2%) completed a postal questionnaire. In addition, a postal

For peer review only - http://bmjopen.bmj.com/site/about/guidelines.xhtml

questionnaire was sent to 126 study members living abroad who remain in contact with the study of whom 83 (65.9%) returned a questionnaire. No attempt was made to contact the remaining 2420 study members: 957 (17.8%) had already died, 620 (11.6%) had previously withdrawn from the study, 448 (8.3%) had emigrated and were no longer in contact with the study, and 395 (7.4%) had been untraceable for more than five years (22).

Relevant ethics approval has been granted for each data collection; ethical approval for the most recent assessment in 2014 was obtained from the Queen Square Research Ethics Committee (14/LO/1073) and the Scotland A Research Ethics Committee (14/SS/1009). Study participants provided written informed consent.

Measurements

Age at puberty

Boys' pubertal stage was based on development of the secondary sexual characteristics which were assessed as part of a school-based medical exam when study members were aged 14-15 (mean age 14.5). Boys were examined for the development of genitalia, voice breaking, pigmented pubic hair and the presence of axillary hair. For the purpose of these analyses, and similar to previous NSHD analyses (10), boys were grouped as prepubescent (infantile genitalia or early adolescent genitalia but no pubic or axillary hair and voice not broken), early puberty (early development of genitalia and some pubic or axillary hair or voice starting to break), advanced puberty (advanced development of genitalia but at least one other indicator not fully mature) or fully mature (advanced development of genitalia, and profuse pubic hair and axillary hair and voice broken). Mothers reported their daughters' age at menarche at the age 15 medical exam. Girls who had not reached menarche at this assessment were asked to recall this information at age 48. In addition to a continuous measure of age at

For peer review only

menarche, girls were also grouped as reaching menarche ≤11 years, 12 years, 13 years or ≥14 years, similar to a previous study examining the association with LTPA (15).

Leisure-time physical activity across adulthood

At ages 36, 43, 53, 60-64 and 68, study participants reported how often they participated in LTPA during nurse interviews or using self-completed questionnaires (6). At age 36, study participants reported the number of times they took part in 27 different sports, exercises and other leisure activities during the previous month using questions based on the Minnesota LTPA questionnaire (7). At age 43, information was collected on participation in sports, exercise or other vigorous leisure activities in the previous year including for how many months and how often in those months activities were performed (6, 8). At ages 53, 60-64 and 68, study participants were asked how often they participated in sports, exercise or other vigorous leisure activities during the previous four weeks. At each age, study participants were classed as active if they reported taking part at least once in LTPA (in the previous month at age 36, per month at age 43, and in the previous 4 weeks at ages 53, 60-64 and 68) or inactive (no LTPA).

Potential confounders

Birth weight, birth order, childhood illness and father's occupational class were identified from the literature to be associated with age at puberty and LTPA and included as model adjustments (23-27). Birth weights were extracted from birth records and information was obtained from mothers on birth order and serious illness in the first 5 years of life (28, 29). Father's Registrar General's occupational class at age 4 (in 1950) (or at age 11 or 15 if missing at age 4 (n=173)) was used to indicate childhood socioeconomic position and was grouped into four categories (I&II: professional, managerial or technical, IIINM: skilled non-manual, IIIM: skilled manual and IV&V: partly skilled or unskilled).

For peer review only - http://bmjopen.bmj.com/site/about/guidelines.xhtml

Statistical analyses

Associations between age at puberty and LTPA across adulthood were examined using standard logistic regression models for LTPA at each age. Models were fit separately for men and women due to differences in the way pubertal timing had been assessed, and expected differences in patterns of association with LTPA. Initial (unadjusted) models were adjusted for birth weight, birth order and childhood illness and additionally for father's occupational class in final models. To formally test whether the association between age at puberty and probability of LTPA changes with increasing age we combined all participants with at least one measure of LTPA and fit mixed-effects logistic regression models (25) containing age at puberty-by-age at LTPA interaction terms. The estimated fixed-effects coefficients were used to plot the log-odds of LTPA for each pubertal-age group against adult age for men and women. Analyses were carried out in STATA 14 (StataCorp, College Station, TX).

RESULTS

Participant characteristics

In total, 1499 men and 1409 women had at least one measure of adulthood LTPA and complete data on age at puberty (pubertal status/menarche) and all selected covariates. A majority of included participants had LTPA data from four of the five different ages in adulthood (a total of 5621 and 5601 LTPA assessments were included in men and women analyses respectively). When compared with those excluded due to missing LTPA data (n=1,596), higher proportions of those with at least one measure of LTPA were female (49.6% vs. 42.5%) and had fathers in occupational classes I & II (23.1% v 20.9%) and lower proportions had low birth weight (4.7% vs. 8.8%) and serious childhood illness (6.5% vs. 17.7%). Over 80% of women had reached menarche by ages 13 years and a quarter of men

For peer review only - http://bmjopen.bmj.com/site/about/guidelines.xhtml

were fully mature at age 15 (Table 1). Higher proportions of men reported taking part in LTPA at ages 36 and 43 but sex-differences were less marked at older ages (Table 1).

Pubertal status at age 15 and leisure-time physical activity across adulthood

Weak associations were found suggesting that earliest maturing boys (those classified as fully mature at age 15) were more likely to participate in LTPA at ages 36 and 43, when compared with boys in advanced and early puberty. At age 43 but not 36, there was also a suggestion that the latest maturing boys were less likely than the earliest maturing boys to participate in LTPA in adulthood (Table 2). No such associations were observed with LTPA at older ages. These findings are supported by evidence that associations changed with age at assessment of LTPA when formally tested using mixed-effects models (P=0.05 for boys' pubertal status-by-age at LTPA interaction). A plot of the log-odds of LTPA at each age in adulthood by pubertal status (estimated from a mixed-effects model) shows small differences between pubertal groups that suggest fully mature boys had the highest likelihood of participating in LTPA at ages 36 and 43 but that differences between pubertal groups reduced with age (Figure 1). Adjustment for birth weight, birth order, childhood illness and father's occupational class had little influence on estimates (Table 2).

Age at menarche and leisure-time physical activity across adulthood

There was no evidence that age at menarche was associated with LTPA across adulthood (Figure 2, Table 3). There was also no evidence of an interaction between age at menarche and age at assessment of LTPA when tested using the mixed-effect models (P for categorical age at menarche-by-age at LTPA interaction=0.9, P for continuous age at menarche-by-age at LTPA interaction=0.7) suggesting that findings did not differ by the age at assessment of LTPA. The ORs of LTPA across adulthood (between 36 and 68 years, estimated with mixed-effects models) for those reaching menarche at 12, 13 and ≥14 years (versus 11 years) were

For peer review only - http://bmjopen.bmj.com/site/about/guidelines.xhtml

1.08 (0.80, 1.47), 1.15 (0.86, 1.54) and 1.15 (0.83, 1.59) respectively (*P*=0.8 for difference between menarche groups).

DISCUSSION

Main findings

This study examined whether timing of puberty, based on clinical assessments of pubertal status at age 15 in boys and prospective reporting of the age at menarche in girls, related to LTPA across adulthood, from ages 36 to 68 years, in the NSHD . There was weak evidence that early maturing boys were more likely to participate in LTPA at younger adult ages and that differences in LTPA between pubertal groups attenuated with increasing age. Age at menarche was not associated with adulthood LTPA.

Comparison with other studies

Very few studies have examined how age at puberty relates to LTPA in adulthood. Largely consistent with our findings are results from the next oldest British birth cohort (1958 NCDS) where the authors reported no associations between stages of boys' axillary hair development and menarche age in girls and LTPA between ages 33-50 years but did not present estimates or test whether associations changed with age (15). Our findings are also largely consistent with null associations reported between self-rated maturity status reported by 12-19 year-old Swedes and subsequent LTPA assessed three times over thirteen years follow-up (14).

In keeping with our findings, Wichstrøm et al. (14) found weak associations between earlier pubertal timing and more LTPA but only at the youngest (mean age=16.5 years) and not the two older follow-up ages to early adulthood (mean ages 21.5 and 28.5 respectively) however, both males and females were included in their analyses so it is unclear if their findings varied

For peer review only

by sex (14). In contrast to our finding that early maturing boys appeared more likely to participate in LTPA at ages 36 and 43, Beunen and colleagues found that later maturity as indicated by later APHV correlated with more participation in sports in 40-year old Belgian men however, APHV was not associated with a leisure-time activities index that excluded sports (16). Finally, that early maturing boys appeared more likely to participate in LTPA at younger adult ages in the NSHD is consistent with findings from adolescent studies that show early maturing boys tend to be more active in sports than their later maturing peers (4, 5).

Explanation of findings

Overall, our findings suggest that age at puberty is not an sizeable correlate of LTPA across adulthood in either men or women. One explanation is that maturity-related variations in the LTPA of adolescents (4-6) may represent transitionary effects on PA which diminish in importance once all peers move beyond puberty into adulthood. Consistent with this, it could be that the differences in LTPA observed in this study between boys in different pubertal groups at age 15 declined with age because some men who were very active as adolescents and young adults (e.g. through frequent involvement in team sports) gave up participation in team sports as they get older and participate at more similar levels to their initially less active peers. This is supported by studies which found late adolescence as a period when many drop out of team sports (30, 31) and findings that chronological age was a more important predictor of PA in older adolescents than pubertal timing (32). Other possible explanations for our findings could include birth cohort differences in associations of pubertal timing with later LTPA, and differences in what LTPA captures in adolescence and adulthood.

Methodological considerations

A strength of this study is that our modelling strategy maximises sample size and improves precision of estimates of association as all individuals with at least one measure of LTPA are

For peer review only - http://bmjopen.bmj.com/site/about/guidelines.xhtml

included under the missing at random assumption (25). Other important strengths included investigation of age-related changes in associations with LTPA, examining association of pubertal timing with adult LTPA separately in males and females and adjustment for prospectively ascertained covariates. In addition, loss to follow-up in NSHD led to only slight differences in characteristics between those included and those with missing data.

There were relatively small numbers of participants in the extreme puberty groups (e.g. <11 for age at menarche in girls) which would have reduced statistical power. This meant it was necessary to combine all those girls who reached menarche by 11 years into a single group (though this is similar to the cut-point used for early menarche in the large UK Biobank study of pubertal timing and adult disease outcomes (9)) and those reaching menarche at or after 14 years into a single group. Since studies show that the extremes of early and late pubertal timing are those at risk of adverse health outcomes (9), it may be that it is those very extreme groups where differences in LTPA are observed and which could not be tested in NSHD due to lower statistical power. However, further investigation revealed no evidence of quadratic effects of age at menarche (P=0.8 for quadratic menarche term from mixed-effects model), and further, parametrising continuous age at menarche using mixed-effects fractional polynomial models also led to similar null findings (all P-values from partial F tests ≥0.9).

LTPA was self-reported therefore, recall and misclassification errors are possible however, self-reports are required to capture activity within specific domains like LTPA (33). Further, in a subsample of this cohort, those participating in LTPA across adulthood were found to spend greater time in moderate-to-vigorous intensity PA as assessed by activity monitors when compared with others reporting no LTPA (34). Finally, our classification of LTPA as at least once per month at each age, which is similar to how we dichotomised LTPA in our recent study (25), may be a limitation of our analysis however, findings were similar when

For peer review only - http://bmjopen.bmj.com/site/about/guidelines.xhtml

using a higher LTPA cut-off of at least five times a month (versus up to 4 times per month) at each adult age.

Conclusions

In a nationally representative British birth cohort study, age at puberty, based on clinical assessments of secondary sexual characteristics in boys and timing of menarche in girls, did not appear to be an important correlate of LTPA across adulthood, reported from ages 36 to 68 years. Age at menarche was not associated with LTPA whereas weak evidence was found that early maturing boys were more likely to participate in LTPA at younger but not older adult ages, and that differences in LTPA by men's pubertal status at age 15 attenuated with increasing adult age. Thus, associations between pubertal timing and adolescent LTPA reported in other studies (4-7, 32, 35), may be transient and lose importance over time and once all peers have transitioned into adulthood.

ACKNOWLEDGMENTS

We thank the study participants for their continuing participation in the MRC National Survey of Health and Development (NSHD). We also thank members of the NSHD scientific and data collection teams who have been involved in the NSHD data collections.

FUNDING

This work was supported by the UK Medical Research Council [programme codes: MC_UU_12019/1]; the funder had no role in the design of the study or the writing of the manuscript and played no part in the decision to submit it for publication.

For peer review only - http://bmjopen.bmj.com/site/about/guidelines.xhtml

CONTRIBUTORSHIP STATEMENT

AE, RC, DB, DK and RH designed the study. AE performed the data analysis and produced the first draft of the manuscript. All authors contributed to the development of the manuscript and read and approved its final version.

COMPETING INTERESTS

The authors declare that they have no competing interests.

DATA SHARING STATEMENT

Data used in this publication are available to bona fide researchers upon request to the NSHD Data Sharing Committee via a standard application procedure. Further details can be found at http://www.nshd.mrc.ac.uk/data. doi: 10.5522/NSHD/Q101; doi: 10.5522/NSHD/Q102; doi: 10.5522/NSHD/Q103.

FIGURE LEGENDS

Figure 1. Log-odds of leisure-time physical activity (LTPA) across adulthood by pubertal status at age 15 years in men from the MRC NSHD, 1946-2014. Includes pubertal status-by-age at LTPA interaction term (*P*=0.05).

Figure 2. Log-odds of leisure-time physical activity across adulthood by age at menarche in women from the MRC NSHD, 1946-2014.

For peer review only - http://bmjopen.bmj.com/site/about/guidelines.xhtml
For peer review only

REFERENCES

1. Patton GC, Viner R. Pubertal transitions in health. Lancet. 2007;369(9567):1130-9.

2. Schooling CM. Life course epidemiology: recognising the importance of puberty. J Epidemiol Community Health. 2015;69(8):820.

3. Telama R. Tracking of physical activity from childhood to adulthood: a review. Obes Facts. 2009;2.

4. Bacil ED, Mazzardo Junior O, Rech CR, Legnani RF, de Campos W. [Physical activity and biological maturation: a systematic review]. Revista paulista de pediatria : orgao oficial da Sociedade de Pediatria de Sao Paulo. 2015;33(1):114-21.

5. Sherar LB, Cumming SP, Eisenmann JC, Baxter-Jones AD, Malina RM. Adolescent biological maturity and physical activity: biology meets behavior. Pediatric exercise science. 2010;22(3):332-49.

6. Cumming SP, Standage M, Gillison FB, Dompier TP, Malina RM. Biological maturity status, body size, and exercise behaviour in British youth: a pilot study. Journal of sports sciences. 2009;27(7):677-86.

7. Davison KK, Werder JL, Trost SG, Baker BL, Birch LL. Why are early maturing girls less active? Links between pubertal development, psychological well-being, and physical activity among girls at ages 11 and 13. Soc Sci Med. 2007;64(12):2391-404.

8. Mrug S, Elliott MN, Davies S, Tortolero SR, Cuccaro P, Schuster MA. Early puberty, negative peer influence, and problem behaviors in adolescent girls. Pediatrics. 2014;133(1):7-14.

9. Day FR, Elks CE, Murray A, Ong KK, Perry JR. Puberty timing associated with diabetes, cardiovascular disease and also diverse health outcomes in men and women: the UK Biobank study. Scientific reports. 2015;5:11208.

For peer review only - http://bmjopen.bmj.com/site/about/guidelines.xhtml

For peer review only

10. Hardy R, Kuh D, Whincup PH, Wadsworth ME. Age at puberty and adult blood pressure and body size in a British birth cohort study. Journal of hypertension. 2006;24(1):59-66.

11. Pierce MB, Kuh D, Hardy R. Role of lifetime body mass index in the association between age at puberty and adult lipids: findings from men and women in a British birth cohort. Ann Epidemiol. 2010;20(9):676-82.

12. Ong KK, Bann D, Wills AK, Ward K, Adams JE, Hardy R, et al. Timing of voice breaking in males associated with growth and weight gain across the life course. The Journal of clinical endocrinology and metabolism. 2012;97(8):2844-52.

13. Kuh D, Muthuri SG, Moore A, Cole TJ, Adams JE, Cooper C, et al. Pubertal timing and bone phenotype in early old age: findings from a British birth cohort study. Int J Epidemiol. 2016.

14. Wichstorm L, Kvalem IL, Soest T. Predictors of growth and decline in leisure-time physical activity from adolescence to adulthood. Health Psychol. 2013;32.

15. Pinto Pereira SM, Li L, Power C. Early-life predictors of leisure-time physical inactivity in midadulthood: findings from a prospective British birth cohort. Am J Epidemiol. 2014;180.

16. Beunen GP, Lefevre J, Philippaerts RM, Delvaux K, Thomis M, Claessens AL. Adolescent correlates of adult physical activity: a 26-year follow-up. Med Sci Sports Exerc. 2004;36.

17. Kuh D, Pierce M, Adams J, Deanfield J, Ekelund U, Friberg P, et al. Cohort profile: updating the cohort profile for the MRC National Survey of Health and Development: a new clinic-based data collection for ageing research. Int J Epidemiol. 2011;40(1):e1-9.

For peer review only - http://bmjopen.bmj.com/site/about/guidelines.xhtml

18. Wadsworth M, Kuh D, Richards M, Hardy R. Cohort Profile: The 1946 National Birth Cohort (MRC National Survey of Health and Development). Int J Epidemiol. 2006;35(1):49-54.

19. Stafford M, Black S, Shah I, Hardy R, Pierce M, Richards M, et al. Using a birth cohort to study ageing: representativeness and response rates in the National Survey of Health and Development. European journal of ageing. 2013;10(2):145-57.

20. Wadsworth M, Butterworth SL, Hardy RJ, Kuh DJ, Richards M, Langenberg C, et al. The life course prospective design: an example of benefits and problems associated with study longevity. Soc Sci Med. 2003;57(11):2193-205.

21. Wadsworth M, Mann SL, Rodgers B, Kuh DJ, Hilder WS, Yusuf EJ. Loss and representativeness in a 43 year follow up of a national birth cohort. J Epidemiol Community Health. 1992;46(3):300-4.

22. Kuh D, Wong A, Shah I, Moore A, Popham M, Curran P, et al. The MRC National Survey of Health and Development reaches age 70: maintaining participation at older ages in a birth cohort study. European Journal of Epidemiology. 2016;31(11):1135-47.

23. Bauman AE, Reis RS, Sallis JF, Wells JC, Loos RJ, Martin BW. Lancet Physical Activity Series Working Group: correlates of physical activity: why are some people physically active and others not? Lancet. 2012;380.

24. Elhakeem A, Cooper R, Bann D, Hardy R. Childhood socioeconomic position and adult leisure-time physical activity: a systematic review. Int J Behav Nutr Phys Act. 2015;12(1):1-27.

25. Elhakeem A, Cooper R, Bann D, Kuh D, Hardy R. Birth Weight, School Sports Ability, and Adulthood Leisure-Time Physical Activity. Med Sci Sports Exerc. 2017;49(1):64-70.

For peer review only - http://bmjopen.bmj.com/site/about/guidelines.xhtml

26. Malina RM BC, Bar-Or O. Biological maturation: concepts and assessment. In: Malina RM BC, Bar-Or O, editor. Growth, maturation, and physical activity. United States: Human Kinetics; 2004. p. 277-306.

27. Mishra GD, Cooper R, Tom SE, Kuh D. Early life circumstances and their impact on menarche and menopause. Women's health (London, England). 2009;5(2):175-90.

28. Kuh DJ, Cooper C. Physical activity at 36 years: patterns and childhood predictors in a longitudinal study. J Epidemiol Community Health. 1992;46.

29. Muthuri SG, Kuh D, Bendayan R, Macfarlane GJ, Cooper R. Chronic physical illness in early life and risk of chronic widespread and regional pain at age 68: evidence from the 1946 British birth cohort. Pain. 2016;157(10):2382-9.

30. Lunn PD. The sports and exercise life-course: a survival analysis of recall data from Ireland. Soc Sci Med. 2010;70(5):711-9.

31. Zick CD, Smith KR, Brown BB, Fan JX, Kowaleski-Jones L. Physical activity during the transition from adolescence to adulthood. Journal of physical activity & health. 2007;4(2):125-37.

32. Finne E, Bucksch J, Lampert T, Kolip P. Age, puberty, body dissatisfaction, and physical activity decline in adolescents. Results of the German Health Interview and Examination Survey (KiGGS). Int J Behav Nutr Phys Act. 2011;8:119.

33. Troiano RP, McClain JJ, Brychta RJ, Chen KY. Evolution of accelerometer methods for physical activity research. Br J Sports Med. 2014;48(13):1019-23.

34. Golubic R, Martin KR, Ekelund U, Hardy R, Kuh D, Wareham N. Levels of physical activity among a nationally representative sample of people in early old age: results of objective and self-reported assessments. Int J Behav Nutr Phys Act. 2014;11.

35. Baker BL, Birch LL, Trost SG, Davison KK. Advanced pubertal status at age 11 and lower physical activity in adolescent girls. The Journal of pediatrics. 2007;151(5):488-93.

For peer review only

Table 1 Descriptive Characteristics by Sex, the Medical Research Council (MRC) National Survey of Health and Development (NSHD), 1946-2014.

	Males (n=1499)	Females (n=1409)	p-value for sex-difference test
Pubertal status at age 15			
fully mature	370 (24.7)		
advanced puberty	457 (30.5)		
early puberty	512 (34.2)		
prepubescent	160 (10.7)		
Age at menarche in years			
≤ 11		238 (16.9)	
12		402 (28.5)	
13		501 (35.6)	
≥ 14		268 (19.0)	
Leisure-time physical activity in previous month (yes v. no) [a]			
age 36	917 (69.1)	730 (56.9)	*P*<0.001
age 43	671 (51.1)	545 (43.6)	*P*<0.001
age 53	614 (52.2)	573 (48.6)	*P*=0.08
age 60-64	294 (34.2)	335 (37.1)	*P*=0.2
age 68	369 (39.1)	401 (40.7)	*P*=0.5
Birth weight (kg)			*P*<0.001
≤ 2.50 (n=129)	54 (3.6)	75 (5.3)	
2.51-3.00 (n=475)	202 (13.5)	273 (194)	
3.01-3.50 (n=1031)	497 (33.2)	534 (37.9)	
3.51-4.00 (n=982)	547 (36.5)	435 (30.9)	
> 4.00 (n=291)	199 (13.3)	92 (6.5)	
Birth order			*P=0.8*
first born (n=1200)	624 (41.6)	576 (40.9)	
second born (n=953)	482 (32.2)	471 (33.4)	
third or later born (n=755)	393 (26.2)	362 (25.7)	
Serious childhood illness			*P=0.2*
no (n=2723)	1395 (93.1)	1328 (94.3)	
yes (n=185)	104 (6.9)	81 (5.8)	
Father's occupational class age 4			*P=0.9*
professional/managerial/technical (n=650)	341 (n=22.8)	309 (21.9)	
skilled non-manual (n=547)	277 (18.5)	270 (19.2)	
skilled manual (n=902)	459 (30.6)	443 (31.4)	
partly skilled or unskilled (n=809)	422 (28.2)	387 (27.5)	

[a] Proportions are for those taking part (at least once per month) in leisure-time physical activity at each age.

For peer review only - http://bmjopen.bmj.com/site/about/guidelines.xhtml

For peer review only

Table 2 Associations between Pubertal Status at Age 15 Years and Leisure-Time Physical Activity (LTPA) at each Adult Age in Men from the MRC NSHD, 1946-2014.

	Odds ratios (95% confidence intervals) of LTPA at least once per month at each adult age versus no LTPA		
	Model 1	Model 2	Model 3
LTPA age 36 years			
fully mature (n=325)	1.00	1.00	1.00
advanced puberty (n=403)	0.69 (0.50, 0.95)	0.70 (0.51, 0.96)	0.69 (0.50, 0.96)
early puberty (n=461)	0.78 (0.57, 1.07)	0.79 (0.58, 1.09)	0.78 (0.57, 1.08)
prepubescent (n=139)	1.01 (0.64, 1.58)	1.03 (0.65, 1.62)	1.05 (0.67, 1.65)
test of association	*P*=0.08	*P*=0.1	*P*=0.07
LTPA age 43 years			
fully mature (n=322)	1.00	1.00	1.00
advanced puberty (n=406)	0.73 (0.55, 0.98)	0.76 (0.56, 1.02)	0.73 (0.54, 0.99)
early puberty (n=445)	0.78 (0.58, 1.04)	0.83 (0.62, 1.11)	0.80 (0.60, 1.08)
prepubescent (n=139)	0.72 (0.48, 1.07)	0.76 (0.50, 1.14)	0.78 (0.52, 1.18)
test of association	*P*=0.2	*P*=0.3	*P*=0.2
LTPA age 53 years			
fully mature (n=292)	1.00	1.00	1.00
advanced puberty (n=359)	1.03 (0.76, 1.41)	1.07 (0.78, 1.46)	1.06 (0.77, 1.45)
early puberty (n=403)	1.10 (0.81, 1.48)	1.14 (0.84, 1.54)	1.12 (0.82, 1.53)
prepubescent (n=122)	1.06 (0.69, 1.62)	1.10 (0.72, 1.69)	1.16 (0.75, 1.79)
test of association	*P*=0.9	*P*=0.9	*P*=0.9
LTPA age 60-64 years			
fully mature (n=217)	1.00 (reference)	1.00 (reference)	1.00 (reference)
advanced puberty (n=263)	1.11 (0.76, 1.62)	1.13 (0.77, 1.65)	1.12 (0.76, 1.65)
early puberty (n=293)	0.96 (0.66, 1.40)	0.97 (0.66, 1.41)	0.98 (0.67, 1.43)
prepubescent (n=88)	0.86 (0.51, 1.48)	0.90 (0.52, 1.54)	0.94 (0.54, 1.63)
test of association	*P*=0.8	*P*=0.8	*P*=0.9
LTPA age 68 years			
fully mature (n=239)	1.00	1.00	1.00
advanced puberty (n=296)	1.03 (0.73, 1.46)	1.04 (0.73, 1.48)	1.03 (0.72, 1.47)
early puberty (n=316)	1.05 (0.74, 1.48)	1.03 (0.73, 1.46)	1.04 (0.73, 1.47)
prepubescent (n=93)	1.01 (0.62, 1.65)	1.01 (0.62, 1.66)	1.06 (0.65, 1.76)
test of association	*P*>0.9	*P*>0.9	*P*>0.9

Analytic samples consist of those with maximum data at each age. Model 1: unadjusted. Model 2: adjusted for birth weight, birth order and childhood illness. Model 3: as for model 2 plus adjustment for father's occupational class. P-values from test of difference between pubertal groups.

For peer review only - http://bmjopen.bmj.com/site/about/guidelines.xhtml

Table 3 Associations between Age at Menarche and Leisure-Time Physical Activity (LTPA) at each Adult Age in Women from the MRC NSHD, 1946-2014.

	Odds ratios (95% confidence intervals) of LTPA at least once per month at each adult age versus no LTPA		
	Model 1	Model 2	Model 3
LTPA age 36 years			
≤ 11 (n=216)	1.00	1.00	1.00
12 (n=363)	0.96 (0.63, 1.45)	0.96 (0.63, 1.45)	1.19 (0.87, 1.62)
13 (n=464)	1.14 (0.76, 1.69)	1.14 (0.76, 1.70)	1.05 (0.74, 1.50)
≥ 14 (n=240)	1.06 (0.67, 1.67)	1.07 (0.67, 1.70)	1.68 (0.88, 3.24)
test of association	*P*=0.8	*P*=0.8	*P*=0.8
LTPA age 43 years			
≤ 11 (n=208)	1.00	1.00	1.00
12 (n=355)	1.16 (0.77, 1.74)	1.17 (0.78, 1.77)	1.27 (0.84, 1.93)
13 (n=440)	1.21 (0.82, 1.78)	1.24 (0.84, 1.84)	1.31 (0.88, 1.96)
≥ 14 (n=248)	1.24 (0.80, 1.92)	1.29 (0.82, 2.02)	1.35 (0.86, 2.13)
test of association	*P*=0.8	*P*=0.7	*P*=0.5
LTPA age 53 years			
≤ 11 (n=203)	1.00	1.00	1.00
12 (n=332)	0.91 (0.61, 1.37)	0.91 (0.61, 1.38)	0.99 (0.65, 1.50)
13 (n=406)	0.82 (0.56, 1.21)	0.84 (0.57, 1.25)	0.90 (0.60, 1.34)
≥ 14 (n=239)	1.03 (0.66, 1.59)	1.05 (0.68, 1.64)	1.10 (0.70, 1.72)
test of association	*P*=0.6	*P*=0.7	*P*=0.8
LTPA age 60-64 years			
≤ 11 (n=148)	1.00	1.00	1.00
12 (n=270)	1.06 (0.68, 1.66)	1.09 (0.69, 1.72)	1.17 (0.74, 1.86)
13 (n=315)	1.24 (0.81, 1.92)	1.31 (0.85, 2.04)	1.39 (0.89, 2.17)
≥ 14 (n=169)	1.01 (0.62, 1.66)	1.06 (0.64, 1.75)	1.09 (0.66, 1.81)
test of association	*P*=0.7	*P*=0.6	*P*=0.5
LTPA age 68 years			
≤ 11 (n=162)	1.00	1.00	1.00
12 (n=280)	0.91 (0.62, 1.36)	0.92 (0.62, 1.38)	1.00 (0.66, 1.50)
13 (n=357)	1.02 (0.70, 1.49)	1.01 (0.69, 1.48)	1.08 (0.73, 1.59)
≥ 14 (n=186)	1.10 (0.72, 1.68)	1.08 (0.70, 1.66)	1.13 (0.73, 1.76)
test of association	*P*=0.8	*P*=0.9	*P*=0.9

Analytic samples consist of those with maximum data at each age. Model 1: unadjusted. Model 2: adjusted for birth weight, birth order and childhood illness. Model 3: as for model 2 plus adjustment for father's occupational class. P-values from test of difference between ages at menarche groups.

For peer review only - http://bmjopen.bmj.com/site/about/guidelines.xhtml

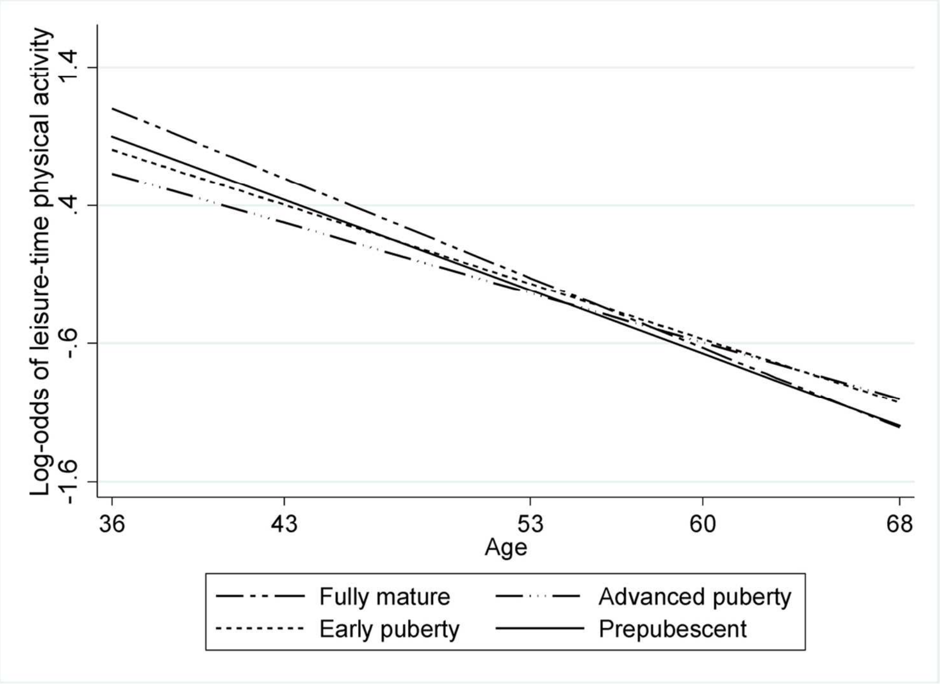

Figure 1. Log-odds of leisure-time physical activity (LTPA) across adulthood by pubertal status at age 15 years in men from the MRC NSHD, 1946-2014. Includes pubertal status-by-age at LTPA interaction term (P=0.05).

115x86mm (300 x 300 DPI)

For peer review only - http://bmjopen.bmj.com/site/about/guidelines.xhtml

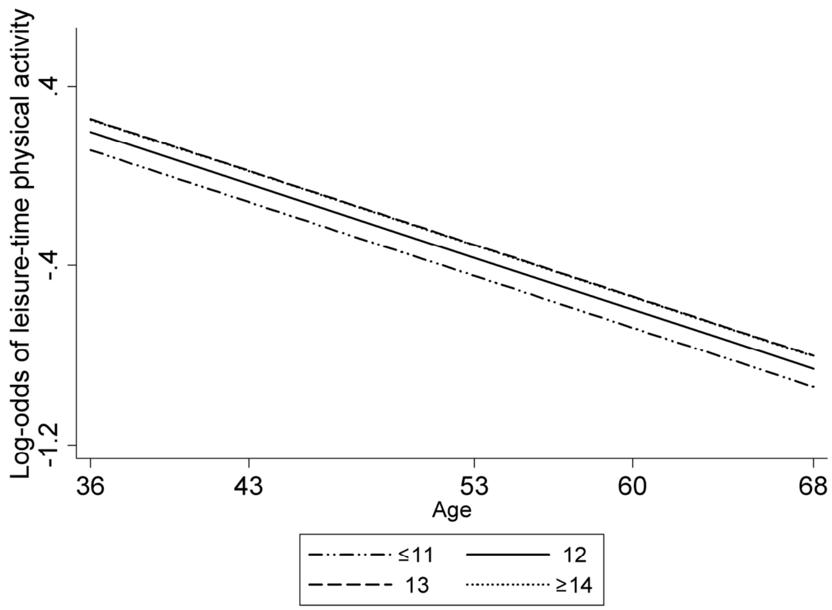

Figure 2. Log-odds of leisure-time physical activity across adulthood by age at menarche in women from the MRC NSHD, 1946-2014.

156x113mm (300 x 300 DPI)

STROBE Guidelines for authors of CORR

To be used by authors of all observational clinical studies published in CORR. For this purpose a cohort study (the term used by STROBE) is considered a longitudinal study typically reporting outcomes of treatment in one or more cohorts; a case-control study is one identifying factors in outcomes; a cross-sectional study is one to identify the prevalence of factors or characteristics in a population at a single point in time.

This table is modified from and used with the permission of The STROBE Initiative, www.strobe-statement.org.

Modifications: We added a fourth column for authors to check inclusion. You must include all items in your manuscript unless the information is not applicable. Information on the study cohort (Items 13 and 14 in the STROBE guidelines) should be provided in Patients and Methods, not in Results; we have omitted the portions of the STROBE guidelines related to Results and Discussion (see our guidelines). The STROBE guidelines were developed for epidemiological studies; "exposed" or "exposure" have been modified with the words "treated" or "treatment."

STROBE Statement—Checklist of items that should be included in reports of ***case-control studies***

	**Item No**	**Recommendation**	**Please insert check where included or N/A where not applicable**
Title and abstract	1	(*a*) Indicate the study's design with a commonly used term in the title or the abstract	1
		(*b*) Provide in the abstract an informative and balanced summary of what was done and what was found	2
		**Introduction**	
Background/rationale	2	Explain the scientific background and rationale for the investigation being reported	4-5
Objectives	3	State specific objectives, including any prespecified hypotheses	5
		**Methods**	
Study design	4	Present key elements of study design early in the paper	5
Setting	5	Describe the setting, locations, and relevant dates, including periods of recruitment, treatment, follow-up, and data collection	5
Participants	6	(*a*) *Cohort study*—Give the eligibility criteria, and the sources and methods of selection of participants. Describe methods of follow-up *Case-control study*—Give the eligibility criteria, and the sources and methods of case	5

For peer review only - http://bmjopen.bmj.com/site/about/guidelines.xhtml

ascertainment and control selection. Give the rationale for the choice of cases and controls<br>*Cross-sectional study*—Give the eligibility criteria, and the sources and methods of selection of participants	
(*b*) *Cohort study*—For matched studies, give matching criteria and number of treated and untreated<br>*Case-control study*—For matched studies, give matching criteria and the number of controls per case	NA

For peer review only

For peer review only - http://bmjopen.bmj.com/site/about/guidelines.xhtml

Participants	13*	(a) Report numbers of individuals at each stage of study—eg numbers potentially eligible, examined for eligibility, confirmed eligible, included in the study, completing follow-up, and analysed	5-6
		(b) Give reasons for non-participation at each stage	5-6
Descriptive data	14*	(a) Give characteristics of study participants (eg demographic, clinical, social) and information on other treatments and potential confounders	8
		(b) Indicate number of participants with missing data for each variable of interest	5-6, 8
		(c) *Cohort study*—Summarise follow-up time (eg, average and total amount)	5-6
Variables	7	Clearly define all outcomes, treatments, predictors, potential confounders, and effect modifiers. Give diagnostic criteria, if applicable	6-8
Data sources/ measurement	8*	For each variable of interest, give sources of data and details of methods of assessment (measurement). Describe comparability of assessment methods if there is more than one group	5-8
Bias	9	Describe any efforts to address potential sources of bias	7-8, 11
Study size	10	Explain how the study size was arrived at	5-8
Quantitative variables	11	Explain how quantitative variables were handled in the analyses. If applicable, describe which groupings were chosen and why	6-8
Statistical methods	12	(*a*) Describe all statistical methods, including those used to control for confounding	7-8
		(*b*) Describe any methods used to examine subgroups and interactions	6-8
		(*c*) Explain how missing data were addressed	6-8
		(*d*) *Cohort study*—If applicable, explain how loss to follow-up was addressed<br>*Case-control study*—If applicable, explain how matching of cases and controls was addressed<br>*Cross-sectional study*—If applicable, describe analytical methods taking account of sampling strategy	7-9
		(*e*) Describe any sensitivity analyses	6-8, 11-12

*Give information separately for cases and controls.

Note: An Explanation and Elaboration article discusses each checklist item and gives methodological background and published examples of transparent reporting. The STROBE checklist is best used in conjunction with this article (freely available on the Web sites of PLoS Medicine at http://www.plosmedicine.org/, Annals of Internal Medicine at http://www.annals.org/, and Epidemiology at http://www.epidem.com/). Information on the STROBE Initiative is available at http://www.strobe-statement.org.

For peer review only - http://bmjopen.bmj.com/site/about/guidelines.xhtml

BMJ Open

Markers of pubertal timing and leisure-time physical activity from ages 36 to 68 years: findings from a British birth cohort

Journal:	*BMJ Open*
Manuscript ID	bmjopen-2017-017407.R1
Article Type:	Research
Date Submitted by the Author:	31-Jul-2017

Complete List of Authors:	Elhakeem, Ahmed; University of Bristol School of Clinical Science,<br>Cooper, Rachel; UCL, MRC Unit for Lifelong Health and Ageing at UCL<br>Bann, David; University College London, UCL Institute of Education<br>Kuh, Diana; MRC Unit for Lifelong Health and Ageing at UCL,<br>Hardy, Rebecca; University College London, MRC Unit for Lifelong Health and Ageing

**Primary Subject Heading**:	Epidemiology
Secondary Subject Heading:	Public health
Keywords:	Life course, leisure-time, physical activity, pubertal timing

SCHOLARONE™
Manuscripts

For peer review only

Markers of pubertal timing and leisure-time physical activity from ages 36 to 68 years: findings from a British birth cohort

Ahmed Elhakeem, PhD[1,2]*, Rachel Cooper, PhD[1], David Bann, PhD[3], Diana Kuh, PhD[1], Rebecca Hardy, PhD[1]

[1]MRC Unit for Lifelong Health and Ageing at UCL, London, UK

[2]Musculoskeletal Research Unit, School of Clinical Sciences, University of Bristol, Bristol, UK

[3] Centre for Longitudinal Studies, UCL Institute of Education, London, UK

*Correspondence: Musculoskeletal Research Unit, School of Clinical Sciences, University of Bristol, Level 1 Learning and Research Building, Southmead hospital, Bristol BS10 5NB, UK, Email: a.elhakeem@bristol.ac.uk, Tel: 0044(0)1174147864

Abstract = 300 words, Text = 3662 words, 40 References, 3 Tables (2 Supplementary files)

For peer review only

ABSTRACT

Objectives

We aimed to examine associations between markers of pubertal timing and leisure-time physical activity (LTPA) from ages 36 to 68 years in men and women from the MRC National Survey of Health and Development.

Study design

Pubertal timing was ascertained by physicians at age 14-15. Boys were grouped, based on their secondary sexual characteristics, as prepubescent, in early stage puberty, advanced stage puberty or fully mature at age 14-15. Girls were grouped as reaching menarche ≤11, 12, 13 or ≥14 years. LTPA was reported at ages 36, 43, 53, 60-64 and 68 and classified as active or inactive at each age. Associations were examined using standard and mixed-effects logistic regression models.

Results

Of 5,362 singleton births recruited, 1499 men and 1409 women had at least one measure of LTPA and data on pubertal timing and selected covariates. When compared with men that were fully mature at age 14-15, those that were in advanced and early stage puberty, but not the prepubescent stage, had lower likelihood of LTPA at younger but not older adult ages (*P*=0.05 for pubertal status-by-age at LTPA interaction in mixed-effects model). For example, fully-adjusted odds ratios (OR) of LTPA (versus no LTPA) at ages 36 and 68 years respectively for advanced puberty versus fully mature were 0.69 (95% confidence intervals: 0.50, 0.96) and 1.03 (0.72, 1.47). Age at menarche was not associated with LTPA (*P*≥0.7 for age at menarche-by-age at LTPA interaction). For example, OR (from mixed-effects model) of LTPA between 36 and 68 years was 1.21 (0.91, 1.61) for menarche at 13 versus ≤11 years.

For peer review only - http://bmjopen.bmj.com/site/about/guidelines.xhtml

Conclusions

In a nationally representative study, there was little evidence to suggest that pubertal timing was an important correlate of LTPA between ages 36 and 68 years. Maturity-related variations in adolescents' LTPA may be transitory and lose importance over time.

KEY WORDS

Life course; leisure-time; physical activity; pubertal timing.

STRENGTHS AND LIMITATIONS OF THIS STUDY

- Assessment of boys' pubertal status at age 14-15 and girls' age at menarche by physicians at school-based medical exams.
- Leisure-time physical activity repeatedly measured prospectively throughout adulthood.
- Limited by fewer numbers of participants at the extremes of pubertal age and lack of information on physical activity participation in childhood and prior to age 36.
- Birth cohort effects and contextual differences in what leisure-time physical activity signifies in adolescence and adulthood may contribute to findings.

For peer review only - http://bmjopen.bmj.com/site/about/guidelines.xhtml

BACKGROUND

Leisure-time physical activity (LTPA) promotes health and therefore it is important to identify groups that might benefit from support to participate in LTPA. Adolescence is a key developmental stage where health behaviours, such as LTPA habits are initiated (1, 2), and which can track into adulthood (3). A major feature of the adolescent period is puberty; a complex process spanning several related domains of growth and development through which adolescents develops into sexually mature adults (4). Besides the importance of adolescence and puberty for the development of health behaviours, deviation from the normal timing of the pubertal process might also influence LTPA (5, 6). Two hypotheses are thought to support this argument; the early maturation hypothesis, which proposes that early maturing adolescents, due to interruptions to their normal course of behavioural development, are at risk of adopting unhealthy behaviour (7), and the maturation deviance hypothesis which suggests that both early and late onset of puberty are associated with subsequent psychosocial problems (8).

Systematic reviews, of mostly cross-sectional studies, suggest that early maturing girls tend to participate less in sports activities than girls who reach their marker of puberty around the average age, thus supporting the early maturation hypothesis (5, 6). Conversely, in males, who are less frequently studied, early maturing boys are generally found to participate more in sports activities than boys reaching their marker of puberty around the average age, favouring the maturation deviance hypothesis (5, 6). While underlying processes are likely complex, it is thought that greater psychosocial problems such as more negative perceptions of their physical appearance and less enjoyment of sports by earlier maturing girls may discourage their participation in sports (5, 6, 9, 10). On the other hand, greater physical strength and perceived competence at sports among earlier maturing boys may encourage

For peer review only - http://bmjopen.bmj.com/site/about/guidelines.xhtml

their participation in sports (5, 6, 9-12). However, it is unclear if maturity-related differences in adolescents LTPA are observed in adults.

Both earlier and later timing of puberty have also been associated with health outcomes in later life that could influence participation in LTPA in adult life. For example, findings from UK Biobank showed both earlier and later ages at puberty were associated with a range of chronic disease outcomes, including heart disease and psychiatric disorders, in both men and women (13). In the MRC National Survey of Health and Development (NSHD), earlier puberty was associated in midlife with greater body mass index (14), higher levels of triglycerides in women (15) and higher blood pressure (14), and greater android fat mass in men (16), whereas later puberty was associated with less bone mineral density (17).

To our knowledge, only three studies have examined associations of age at puberty with LTPA in adults. Perceived relative pubertal status reported by 12-19-year-old Norwegians was not related to LTPA over thirteen years of follow-up in one study (18), and in the 1958 National Child Development Study (NCDS), age at puberty was unrelated to midlife LTPA (19). However, among 166 40-year old Belgian men (20), later age at peak height velocity (APHV), a marker of later pubertal onset, correlated with higher participation in sports. It is possible that associations between pubertal timing and LTPA weaken with age, as the pathways on which puberty acts become less important influences on LTPA, or possibly due to health conditions associated with early or late puberty being associated with declines or improvement in health and LTPA. Therefore, detailed studies with repeated measures of LTPA across adult life are needed, including those that examine associations in both male and female samples. This study examines associations of age at menarche in women and pubertal stage at age 14-15 in men with LTPA from ages 36 to 68 years, and whether any associations found change with age at assessment of LTPA.

For peer review only - http://bmjopen.bmj.com/site/about/guidelines.xhtml

METHODS

Participants

The NSHD is a nationally representative sample of 5,362 British singleton births during one week in March 1946 and followed up regularly across childhood and adulthood (21, 22). In childhood and adolescence, data on NSHD participants were collected from parents, health care and educational professionals and study participants themselves. At age 14-15 (1961), medical examinations were carried out by school doctors. At ages 36 (1982), 43 (1989) and 53 (1999) trained nurses interviewed and assessed the study participants in their own homes. At age 60-64 (2006-2010) they attended one of 6 clinical research facilities or received a home visit, and at age 68 (2014) they completed a postal questionnaire followed by a home visit.

Of those successfully contacted at ages 36 (n=3322), 43 (n=3262), 53 (n=3035), 60-64 (n=2661) and 68 years (n=2453), 99.6%, 100%, 98.4%, 82.2% and 99.1% respectively provided information on LTPA (3766 participants had at least one measure of LTPA). The participating samples at ages 43, 53 and 60-64 have been found to be broadly representative of similar aged members of the general UK population (23-25). At the most recent round of data collection at age 68, of the 2816 people in the target sample living in England, Scotland and Wales, 2370 (84.2%) completed a postal questionnaire. In addition, a postal questionnaire was sent to 126 study members living abroad who remain in contact with the study of whom 83 (65.9%) returned a questionnaire. No attempt was made to contact the remaining 2420 study members: 957 (17.8%) had already died, 620 (11.6%) had previously withdrawn from the study, 448 (8.3%) had emigrated and were no longer in contact with the study, and 395 (7.4%) had been untraceable for more than five years (26).

For peer review only - http://bmjopen.bmj.com/site/about/guidelines.xhtml

Relevant ethics approval has been granted for each data collection; ethical approval for the most recent assessment in 2014 was obtained from the Queen Square Research Ethics Committee (14/LO/1073) and the Scotland A Research Ethics Committee (14/SS/1009). Study participants provided written informed consent.

Measurements

Pubertal measurements

In 1961, when study members were aged 14-15 (mean age 14.5), they (both boys and girls) were assessed as part of a school-based medical exam carried out in school clinics by physicians with a parent present (14, 15, 17, 24). These physical examinations, which predated Tanner staging, included assessments of the visibility of pigmented pubic hair (none, sparse, profuse), visibility of axillary hair (no, yes) and, for boys only, development of genitalia (infantile, early, advanced), and whether the voice had broken (no, starting, completely broken). On the basis of these four indicators of secondary sexual characteristics, boys were grouped as prepubescent (infantile genitalia or early adolescent genitalia but no pubic or axillary hair and voice not broken), early stage puberty (early development of genitalia and some pubic or axillary hair or voice starting to break), advanced stage puberty (advanced development of genitalia but at least one other indicator not fully mature) or fully mature (advanced development of genitalia, and profuse pubic hair and axillary hair and voice broken) at age 14-15.

Mothers reported their daughters' age at menarche at the age 14-15 school-based medical exam. In addition to a continuous measure of age at menarche, women were also grouped as reaching menarche ≤11 years, 12 years, 13 years or ≥14 years, similar to a previous study examining the association with LTPA (19). All women were asked to recall their age at menarche at age 48. Women that had not reached menarche by the age 14-15 medical exam

For peer review only - http://bmjopen.bmj.com/site/about/guidelines.xhtml

and who subsequently reported reaching menarche at an age that was consistent with this (i.e. at/after age 14-15) were included (in the ≥14 years group). Recalled age at menarche was not used to replace values if they were missing from mother's report due to evidence that recall of age at menarche may not be valid (27). For these analyses, we use pubertal status at age 14-15 in males and age at menarche in females as markers of pubertal timing (4).

Leisure-time physical activity from ages 36 to 68 years

At ages 36, 43, 53, 60-64 and 68, study participants reported how often they participated in LTPA during nurse interviews or using self-completed questionnaires (6). At age 36, study participants reported the number of times they took part in 27 different sports, exercises and other leisure activities during the previous month using questions based on the Minnesota LTPA questionnaire (7). At age 43, information was collected on participation in sports, exercise or other vigorous leisure activities in the previous year including for how many months and how often in those months activities were performed (6, 8). At ages 53, 60-64 and 68, study participants were asked how often they participated in sports, exercise or other vigorous leisure activities during the previous four weeks. At each age, study participants were classed as active if they reported taking part at least once in LTPA (in the previous month at age 36, per month at age 43, and in the previous 4 weeks at ages 53, 60-64 and 68) or inactive (no LTPA).

Potential confounders

Potential confounders were selected *a priori.* These were birth weight, birth order, childhood illness and father's occupational class, all of which had been identified from the literature as being associated with both pubertal timing and LTPA (28-32). Birth weights were extracted from birth records and information was obtained from mothers on birth order and serious illness in the first 5 years of life (33, 34). Father's Registrar General's occupational class at

For peer review only - http://bmjopen.bmj.com/site/about/guidelines.xhtml

For peer review only

age 4 (in 1950) (or at age 11 or 15 if missing at age 4 (n=173)) was used to indicate childhood socioeconomic position and was grouped into four categories (I&II: professional, managerial or technical, IIINM: skilled non-manual, IIIM: skilled manual and IV&V: partly skilled or unskilled).

Statistical analyses

Models were fit separately for men and women due to differences in the way pubertal timing had been assessed, and expected differences in patterns of association with LTPA (5, 6). Associations between women's age at menarche and men's pubertal stage at 14-15 and LTPA were examined using standard logistic regression models for LTPA at each age from 36 to 68 years. Initial (unadjusted) models (Model 1) were adjusted for birth weight, birth order and childhood illness (Model 2) and additionally for father's occupational class in final models (Model 3).

To examine whether any associations were mediated by health in adulthood, final models were refitted with additional adjustment for physical health status at age 36. Physical health was derived from information collected during nurse interviews at age 36 years on weight, disability, self-reported health problems, hypertension, lung function and incidence of hospital admissions (35). This information was used to categorise participants into worst, intermediate or best physical health (35). To formally test whether the association between pubertal timing and probability of LTPA changes with increasing age we combined all participants with at least one measure of LTPA and fit mixed-effects logistic regression models (25) containing pubertal timing-by-age at LTPA interaction terms. These models were unadjusted and fitted with random intercepts and slopes for age at assessment of LTPA and an independent correlation structure. An assumption of linearity was used for examining

For peer review only - http://bmjopen.bmj.com/site/about/guidelines.xhtml

whether associations change with age at assessment of LTPA. All analyses were carried out in STATA 14 (StataCorp, College Station, TX).

RESULTS

Participant characteristics

In total, 1499 men and 1409 women had at least one measure of adulthood LTPA and complete data on age at puberty (pubertal status/menarche) and all selected covariates. A majority of included participants had LTPA data from four of the five different ages in adulthood (a total of 5621 and 5601 LTPA assessments were included in men and women analyses respectively). When compared with those excluded due to missing LTPA data (n=1,596), higher proportions of those with at least one measure of LTPA were female (49.6% vs. 42.5%) and had fathers in occupational classes I & II (23.1% v 20.9%) and lower proportions had low birth weight (4.7% vs. 8.8%) and serious childhood illness (6.5% vs. 17.7%). Over 80% of women had reached menarche by ages 13 years and a quarter of men were fully mature at age 15 (Table 1). Higher proportions of men reported taking part in LTPA at ages 36 and 43 but sex-differences were less marked at older ages (Table 1).

Men: pubertal status at age 15 and LTPA from 36 to 68 years

Weak associations were found suggesting that the earliest maturing boys (i.e. those classified as fully mature at age 15) were more likely to participate in LTPA at ages 36 and 43, when compared with boys in advanced and early stage puberty at age 15. At age 43 but not 36, there was also a suggestion that the latest maturing boys were less likely than the earliest maturing boys to participate in LTPA (Table 2). No such associations were observed with LTPA at older ages. Adjustment for birth weight, birth order, childhood illness and father's occupational class had little influence on estimates (Table 2). These findings are supported by evidence that associations changed with age at assessment of LTPA when formally tested

For peer review only - http://bmjopen.bmj.com/site/about/guidelines.xhtml

For peer review only

using mixed-effects models (P=0.05 for boys' pubertal status-by-age at LTPA interaction). Further adjustment for physical health status at age 36 had little influence on estimates (supplementary table 1).

Women: age at menarche and LTPA from 36 to 68 years

There was no evidence that age at menarche was associated with LTPA between 36 to 68 years (Table 3). There was also no evidence of an interaction between age at menarche and age at assessment of LTPA when tested using the mixed-effect models (P for categorical age at menarche-by-age at LTPA interaction=0.9, P for continuous age at menarche-by-age at LTPA interaction=0.7) suggesting that findings did not differ by the age at assessment of LTPA. The ORs of LTPA between 36 and 68 years (estimated with mixed-effects models) for those reaching menarche at 12, 13 and ≥14 years (versus 11 years) were 1.08 (0.80, 1.47), 1.15 (0.86, 1.54) and 1.15 (0.83, 1.59) respectively (P=0.8 for difference between menarche groups). Further adjustment for physical health status at age 36 did not influence estimates (supplementary table 2).

DISCUSSION

Main findings

This study examined whether markers of pubertal timing in males and females (clinical assessments of men's pubertal status based on developmental stages of secondary sexual characteristics at age 14-15 and prospective reporting of women's age at menarche) were related to LTPA from ages 36 to 68 years in the MRC NSHD (1946 British birth cohort). There was weak evidence that, when compared with the earliest maturing boys (i.e. fully mature at age 14-15), those in advanced and early stages of puberty, but not the prepubescent stage, were less likely to participate in LTPA at ages 36 and 43, and that differences in LTPA

For peer review only - http://bmjopen.bmj.com/site/about/guidelines.xhtml

by pubertal status attenuated with increasing age. Age at menarche was not associated with adulthood LTPA.

Comparison with other studies

Very few studies have examined how markers of pubertal timing relate to LTPA in adulthood. Largely consistent with our findings are results from the next oldest British birth cohort (1958 NCDS) where the authors reported no associations between stages of boys' axillary hair development and menarche age in girls and LTPA between ages 33-50 years but did not present estimates or test whether associations changed with age (19). Our findings are also largely consistent with null associations reported between self-rated maturity status reported by 12-19 year-old Swedes and subsequent LTPA assessed three times over thirteen years follow-up (18).

In keeping with our findings, Wichstrøm et al. (18) found weak associations between earlier pubertal timing and more LTPA but only at the youngest (mean age=16.5 years) and not the two older follow-up ages to early adulthood (mean ages 21.5 and 28.5 respectively) however, both males and females were included in their analyses so it is unclear if their findings varied by sex (18). In contrast to our finding that early maturing boys appeared more likely to participate in LTPA at ages 36 and 43, Beunen and colleagues (2004) found that later maturity as indicated by later APHV correlated with more participation in sports in 40-year old Belgian men however, APHV was not associated with a leisure-time activities index that excluded sports (20). Finally, that early maturing boys appeared more likely to participate in LTPA at younger adult ages in the NSHD is consistent with findings from adolescent studies that show early maturing boys tend to be more active in sports than their later maturing peers (5, 6).

Explanation of findings

For peer review only - http://bmjopen.bmj.com/site/about/guidelines.xhtml

Overall, our findings suggest that pubertal timing is not a sizeable correlate of LTPA across adulthood (from 36 to 68 years) in either men or women. One explanation is that maturity-related variations in the LTPA of adolescents (5, 6, 11) may represent transitionary effects on PA which diminish in importance once all peers move beyond puberty into adulthood. Consistent with this, it could be that the differences in LTPA observed in this study between boys in different pubertal groups at age 15 declined with age because some men who were very active as adolescents and young adults (e.g. through frequent involvement in team sports) reduced their participation in team sports as they get older. This is supported by studies which found late adolescence as a period when many drop out of team sports (36, 37) and findings that chronological age was a more important predictor of PA in older adolescents than pubertal timing (38). Other possible explanations for our findings could include birth cohort differences in associations of pubertal timing with later LTPA, and differences in what LTPA captures in adolescence and adulthood.

Methodological considerations

This study benefits from an investigation of age-related changes in associations of pubertal timing with LTPA, where we included all individuals with at least one measure of LTPA under the missing at random assumption, examining association separately in males and females and adjustment for prospectively ascertained covariates. In addition, loss to follow-up in NSHD led to only slight differences in characteristics between those included and those with missing data. The prospective ascertainment by school-based physicians of men's pubertal status at age 14-15 and women's age at menarche is another notable strength.

Limitations include relatively small numbers of participants in the extreme puberty groups (e.g. <11 for age at menarche in girls) which would have reduced statistical power. This meant it was necessary to combine all those girls who reached menarche by 11 years into a

For peer review only - http://bmjopen.bmj.com/site/about/guidelines.xhtml

single group (though this is similar to the cut-point used for early menarche in the large UK Biobank study of pubertal timing and adult disease outcomes (9)) and those reaching menarche at or after 14 years into a single group. Since studies show that the extremes of early and late pubertal timing are those at risk of adverse health outcomes (13), it may be that it is those very extreme groups where differences in LTPA are observed and which could not be tested in NSHD due to lower statistical power. However, further investigation revealed no evidence of quadratic effects of age at menarche (P=0.8 for quadratic menarche term from mixed-effects model), and further, parametrising continuous age at menarche using mixed-effects fractional polynomial models also led to similar null findings (all P-values from partial F tests ≥0.9).

While the markers of pubertal timing used here were prospectively assessed, some misclassification in the categories used may still be possible. In addition, we speculated that adult health may mediate associations between puberty timing and LTPA however, it is likely that there is a bidirectional process such that LTPA also mediates associations between pubertal timing and adult health. Further, the markers of pubertal timing used were not directly comparable for males and females, and the historical nature of these assessments meant we did not have data on other markers of puberty such as Tanner staging.

LTPA was self-reported therefore, recall and misclassification errors are possible however, self-reports are required to capture activity within specific domains like LTPA (39). Further, in a subsample of this cohort, those participating in LTPA across adulthood were found to spend greater time in moderate-to-vigorous intensity PA as assessed by activity monitors when compared with others reporting no LTPA (40). In addition, our classification of LTPA as at least once per month at each age, which is similar to how we dichotomised LTPA in our recent study (25), may be a limitation given it summarised activity according to whether or not any participation in LTPA was reported, however, findings were similar when using a

For peer review only - http://bmjopen.bmj.com/site/about/guidelines.xhtml

higher LTPA cut-off of at least five times a month (versus up to 4 times per month) at each adult age. Further, that the LTPA measures were dichotomous in nature limits the amount of LTPA information used however, this does provide comparable measures to allow tracking of LTPA over 32 years. Additionally, we only examined LTPA in this study and other activity domains (e.g. active transport) may be important for overall PA levels. Furthermore, while we adjusted for several early life confounders, we did not have data on early life LTPA which may be important given the tracking of LTPA across life, and it is possible that maturity-related variations in LTPA may be less pronounced in those with a high level of sports during childhood (6). Finally, residual confounding due to unmeasured confounders and measurement error in the measured confounders could influence findings.

Conclusions

In a nationally representative prospective British birth cohort study, pubertal timing, based on clinical assessments of stages of secondary sexual characteristics at age 14-15 in boys and the age at menarche in girls, did not appear to be an important correlate of LTPA reported from ages 36 to 68 years. Age at menarche was not associated with LTPA whereas weak evidence was found that, when compared with the earliest maturing boys, those in advanced and early stages of puberty, but not prepubescent stage, were less likely to participate in LTPA at younger but not older adult ages, and that differences in LTPA by men's pubertal status at age 14-15 attenuated with increasing adult age. Thus, associations between pubertal timing and adolescent LTPA reported in other studies (5, 6, 10, 11, 38), may be transient and lose importance over time and once all peers have transitioned into adulthood.

ACKNOWLEDGMENTS

For peer review only - http://bmjopen.bmj.com/site/about/guidelines.xhtml

We thank the study participants for their continuing participation in the MRC National Survey of Health and Development (NSHD). We also thank members of the NSHD scientific and data collection teams who have been involved in the NSHD data collections.

FUNDING

This work was supported by the UK Medical Research Council [programme codes: MC_UU_12019/1]; the funder had no role in the design of the study or the writing of the manuscript and played no part in the decision to submit it for publication.

CONTRIBUTORSHIP STATEMENT

AE, RC, DB, DK and RH designed the study. AE performed the data analysis and produced the first draft of the manuscript. All authors contributed to the development of the manuscript and read and approved its final version.

COMPETING INTERESTS

The authors declare that they have no competing interests.

DATA SHARING STATEMENT

Data used in this publication are available to bona fide researchers upon request to the NSHD Data Sharing Committee via a standard application procedure. Further details can be found at http://www.nshd.mrc.ac.uk/data. doi: 10.5522/NSHD/Q101; doi: 10.5522/NSHD/Q102; doi: 10.5522/NSHD/Q103.

For peer review only - http://bmjopen.bmj.com/site/about/guidelines.xhtml

REFERENCES

1. Patton GC, Viner R. Pubertal transitions in health. Lancet. 2007;369(9567):1130-9.

2. Schooling CM. Life course epidemiology: recognising the importance of puberty. J Epidemiol Community Health. 2015;69(8):820.

3. Telama R. Tracking of physical activity from childhood to adulthood: a review. Obes Facts. 2009;2(3):187-95.

4. Dorn LD, Dahl RE, Woodward HR, Biro F. Defining the boundaries of early adolescence: a user's guide to assessing pubertal status and pubertal timing in research with adolescents. Appl Dev Sci. 2006;10(1):30-56.

5. Bacil ED, Mazzardo Junior O, Rech CR, Legnani RF, de Campos W. [Physical activity and biological maturation: a systematic review]. Rev Paul Pediatr. 2015;33(1):114-21.

6. Sherar LB, Cumming SP, Eisenmann JC, Baxter-Jones AD, Malina RM. Adolescent biological maturity and physical activity: biology meets behavior. Pediatr Exerc Sci. 2010;22(3):332-49.

7. Petersen A, Taylor B, Aldelson J. The biological approach to adolescence: Biological change and psychosocial adaptation. Handbook of adolescent psychology. New York: Wiley; 1980. p. 117–55.

8. Alasker F. Timing of puberty and reactions to pubertal changes. In: Rutter M, editor. Psychosocial disurbances in young people. Cambridge: Cambridge University Press; 1995. p. 37-82.

9. Lee EY, An K, Jeon JY, Rodgers WM, Harber VJ, Spence JC. Biological maturation and physical activity in South Korean adolescent girls. Med Sci Sports Exerc. 2016;48(12):2454-61.

For peer review only - http://bmjopen.bmj.com/site/about/guidelines.xhtml

10. Davison KK, Werder JL, Trost SG, Baker BL, Birch LL. Why are early maturing girls less active? Links between pubertal development, psychological well-being, and physical activity among girls at ages 11 and 13. Soc Sci Med. 2007;64(12):2391-404.

11. Cumming SP, Standage M, Gillison FB, Dompier TP, Malina RM. Biological maturity status, body size, and exercise behaviour in British youth: a pilot study. J Sports Sci. 2009;27(7):677-86.

12. Mrug S, Elliott MN, Davies S, Tortolero SR, Cuccaro P, Schuster MA. Early puberty, negative peer influence, and problem behaviors in adolescent girls. Pediatrics. 2014;133(1):7-14.

13. Day FR, Elks CE, Murray A, Ong KK, Perry JR. Puberty timing associated with diabetes, cardiovascular disease and also diverse health outcomes in men and women: the UK Biobank study. Sci Rep. 2015;5:11208.

14. Hardy R, Kuh D, Whincup PH, Wadsworth ME. Age at puberty and adult blood pressure and body size in a British birth cohort study. J Hypertens. 2006;24(1):59-66.

15. Pierce MB, Kuh D, Hardy R. Role of lifetime body mass index in the association between age at puberty and adult lipids: findings from men and women in a British birth cohort. Ann Epidemiol. 2010;20(9):676-82.

16. Ong KK, Bann D, Wills AK, Ward K, Adams JE, Hardy R, et al. Timing of voice breaking in males associated with growth and weight gain across the life course. J Clin Endocrinol Metab. 2012;97(8):2844-52.

17. Kuh D, Muthuri SG, Moore A, Cole TJ, Adams JE, Cooper C, et al. Pubertal timing and bone phenotype in early old age: findings from a British birth cohort study. Int J Epidemiol. 2016.

18. Wichstorm L, Kvalem IL, Soest T. Predictors of growth and decline in leisure-time physical activity from adolescence to adulthood. Health Psychol. 2013;32(7):775-84.

For peer review only - http://bmjopen.bmj.com/site/about/guidelines.xhtml

For peer review only

19. Pinto Pereira SM, Li L, Power C. Early-life predictors of leisure-time physical inactivity in midadulthood: findings from a prospective British birth cohort. Am J Epidemiol. 2014;180(11):1098-108.

20. Beunen GP, Lefevre J, Philippaerts RM, Delvaux K, Thomis M, Claessens AL. Adolescent correlates of adult physical activity: a 26-year follow-up. Med Sci Sports Exerc. 2004;36(11):1930-6.

21. Kuh D, Pierce M, Adams J, Deanfield J, Ekelund U, Friberg P, et al. Cohort profile: updating the cohort profile for the MRC National Survey of Health and Development: a new clinic-based data collection for ageing research. Int J Epidemiol. 2011;40(1):e1-9.

22. Wadsworth M, Kuh D, Richards M, Hardy R. Cohort Profile: The 1946 National Birth Cohort (MRC National Survey of Health and Development). Int J Epidemiol. 2006;35(1):49-54.

23. Stafford M, Black S, Shah I, Hardy R, Pierce M, Richards M, et al. Using a birth cohort to study ageing: representativeness and response rates in the National Survey of Health and Development. Eur J Ageing. 2013;10(2):145-57.

24. Wadsworth M, Butterworth SL, Hardy RJ, Kuh DJ, Richards M, Langenberg C, et al. The life course prospective design: an example of benefits and problems associated with study longevity. Soc Sci Med. 2003;57(11):2193-205.

25. Wadsworth M, Mann SL, Rodgers B, Kuh DJ, Hilder WS, Yusuf EJ. Loss and representativeness in a 43 year follow up of a national birth cohort. J Epidemiol Community Health. 1992;46(3):300-4.

26. Kuh D, Wong A, Shah I, Moore A, Popham M, Curran P, et al. The MRC National Survey of Health and Development reaches age 70: maintaining participation at older ages in a birth cohort study. Eur J Epidemiol. 2016;31(11):1135-47.

For peer review only

27. Cooper R, Blell M, Hardy R, Black S, Pollard TM, Wadsworth ME, et al. Validity of age at menarche self-reported in adulthood. J Epidemiol Community Health. 2006;60(11):993-7.

28. Bauman AE, Reis RS, Sallis JF, Wells JC, Loos RJ, Martin BW. Lancet Physical Activity Series Working Group: correlates of physical activity: why are some people physically active and others not? Lancet. 2012;380(9838):258-71.

29. Elhakeem A, Cooper R, Bann D, Hardy R. Childhood socioeconomic position and adult leisure-time physical activity: a systematic review. Int J Behav Nutr Phys Act. 2015;12(1):1-27.

30. Elhakeem A, Cooper R, Bann D, Kuh D, Hardy R. Birth weight, school sports ability, and adulthood leisure-time physical activity. Med Sci Sports Exerc. 2017;49(1):64-70.

31. Malina RM BC, Bar-Or O. Biological maturation: concepts and assessment. In: Malina RM BC, Bar-Or O, editor. Growth, maturation, and physical activity. United States: Human Kinetics; 2004. p. 277-306.

32. Mishra GD, Cooper R, Tom SE, Kuh D. Early life circumstances and their impact on menarche and menopause. Womens Health (Lond). 2009;5(2):175-90.

33. Kuh DJ, Cooper C. Physical activity at 36 years: patterns and childhood predictors in a longitudinal study. J Epidemiol Community Health. 1992;46(2):114-9.

34. Muthuri SG, Kuh D, Bendayan R, Macfarlane GJ, Cooper R. Chronic physical illness in early life and risk of chronic widespread and regional pain at age 68: evidence from the 1946 British birth cohort. Pain. 2016;157(10):2382-9.

35. Kuh DJ, Wadsworth ME. Physical health status at 36 years in a British national birth cohort. Soc Sci Med. 1993;37(7):905-16.

36. Lunn PD. The sports and exercise life-course: a survival analysis of recall data from Ireland. Soc Sci Med. 2010;70(5):711-9.

For peer review only

37. Zick CD, Smith KR, Brown BB, Fan JX, Kowaleski-Jones L. Physical activity during the transition from adolescence to adulthood. J Phys Act Health. 2007;4(2):125-37.

38. Finne E, Bucksch J, Lampert T, Kolip P. Age, puberty, body dissatisfaction, and physical activity decline in adolescents. Results of the German Health Interview and Examination Survey (KiGGS). Int J Behav Nutr Phys Act. 2011;8:119.

39. Troiano RP, McClain JJ, Brychta RJ, Chen KY. Evolution of accelerometer methods for physical activity research. Br J Sports Med. 2014;48(13):1019-23.

40. Golubic R, Martin KR, Ekelund U, Hardy R, Kuh D, Wareham N. Levels of physical activity among a nationally representative sample of people in early old age: results of objective and self-reported assessments. Int J Behav Nutr Phys Act. 2014;11.

For peer review only

For peer review only - http://bmjopen.bmj.com/site/about/guidelines.xhtml

Table 1 Descriptive Characteristics by Sex, the Medical Research Council (MRC) National Survey of Health and Development (NSHD), 1946-2014.

	Males (n=1499)	Females (n=1409)	p-value for sex-difference test
Pubertal status at age 14-15			
fully mature	370 (24.7)		
advanced stage puberty	457 (30.5)		
early stage puberty	512 (34.2)		
prepubescent	160 (10.7)		
Age at menarche in years			
≤ 11		238 (16.9)	
12		402 (28.5)	
13		501 (35.6)	
≥ 14		268 (19.0)	
Leisure-time physical activity in previous month (yes v. no) [a]			
age 36	917 (69.1)	730 (56.9)	*P*<0.001
age 43	671 (51.1)	545 (43.6)	*P*<0.001
age 53	614 (52.2)	573 (48.6)	*P*=0.08
age 60-64	294 (34.2)	335 (37.1)	*P*=0.2
age 68	369 (39.1)	401 (40.7)	*P*=0.5
Birth weight (kg)			*P*<0.001
≤ 2.50 (n=129)	54 (3.6)	75 (5.3)	
2.51-3.00 (n=475)	202 (13.5)	273 (194)	
3.01-3.50 (n=1031)	497 (33.2)	534 (37.9)	
3.51-4.00 (n=982)	547 (36.5)	435 (30.9)	
> 4.00 (n=291)	199 (13.3)	92 (6.5)	
Birth order			*P=0.8*
first born (n=1200)	624 (41.6)	576 (40.9)	
second born (n=953)	482 (32.2)	471 (33.4)	
third or later born (n=755)	393 (26.2)	362 (25.7)	
Serious childhood illness			*P=0.2*
no (n=2723)	1395 (93.1)	1328 (94.3)	
yes (n=185)	104 (6.9)	81 (5.8)	
Father's occupational class age 4			*P=0.9*
professional/managerial/technical (n=650)	341 (n=22.8)	309 (21.9)	
skilled non-manual (n=547)	277 (18.5)	270 (19.2)	
skilled manual (n=902)	459 (30.6)	443 (31.4)	
partly skilled or unskilled (n=809)	422 (28.2)	387 (27.5)	

[a] Proportions are for those taking part (at least once per month) in leisure-time physical activity at each age.

For peer review only - http://bmjopen.bmj.com/site/about/guidelines.xhtml

Table 2 Associations between Pubertal Status at Age 14-15 Years and Leisure-Time Physical Activity (LTPA) at each Adult Age in Men from the MRC NSHD, 1946-2014.

	N (%) LTPA*	Odds ratios (95% confidence intervals) of LTPA at least once per month at each adult age versus no LTPA		
		Model 1	Model 2	Model 3
LTPA age 36 years				
fully mature (n=325)	238 (73.2)	1.00	1.00	1.00
advanced stage puberty (n=403)	263 (65.3)	0.69 (0.50, 0.95)	0.70 (0.51, 0.96)	0.69 (0.50, 0.96)
early stage puberty (n=461)	314 (68.1)	0.78 (0.57, 1.07)	0.79 (0.58, 1.09)	0.78 (0.57, 1.08)
prepubescent (n=139)	102 (73.4)	1.01 (0.64, 1.58)	1.03 (0.65, 1.62)	1.05 (0.67, 1.65)
test of association		*P*=0.08	*P*=0.1	*P*=0.07
LTPA age 43 years				
fully mature (n=322)	182 (56.5)	1.00	1.00	1.00
advanced stage puberty (n=406)	198 (48.8)	0.73 (0.55, 0.98)	0.76 (0.56, 1.02)	0.73 (0.54, 0.99)
early stage puberty (n=445)	224 (50.3)	0.78 (0.58, 1.04)	0.83 (0.62, 1.11)	0.80 (0.60, 1.08)
prepubescent (n=139)	67 (48.2)	0.72 (0.48, 1.07)	0.76 (0.50, 1.14)	0.78 (0.52, 1.18)
test of association		*P*=0.2	*P*=0.3	*P*=0.2
LTPA age 53 years				
fully mature (n=292)	149 (51.0)	1.00	1.00	1.00
advanced stage puberty (n=359)	186 (51.8)	1.03 (0.76, 1.41)	1.07 (0.78, 1.46)	1.06 (0.77, 1.45)
early stage puberty (n=403)	215 (53.4)	1.10 (0.81, 1.48)	1.14 (0.84, 1.54)	1.12 (0.82, 1.53)
prepubescent (n=122)	64 (52.5)	1.06 (0.69, 1.62)	1.10 (0.72, 1.69)	1.16 (0.75, 1.79)
test of association		*P*=0.9	*P*=0.9	*P*=0.9
LTPA age 60-64 years				
fully mature (n=217)	74 (34.1)	1.00 (reference)	1.00 (reference)	1.00 (reference)
advanced stage puberty (n=263)	96 (36.5)	1.11 (0.76, 1.62)	1.13 (0.77, 1.65)	1.12 (0.76, 1.65)
early stage puberty (n=293)	97 (33.1)	0.96 (0.66, 1.40)	0.97 (0.66, 1.41)	0.98 (0.67, 1.43)
prepubescent (n=88)	27 (30.7)	0.86 (0.51, 1.48)	0.90 (0.52, 1.54)	0.94 (0.54, 1.63)
test of association		*P*=0.8	*P*=0.8	*P*=0.9
LTPA age 68 years				
fully mature (n=239)	92 (38.5)	1.00	1.00	1.00
advanced stage puberty (n=296)	116 (39.2)	1.03 (0.73, 1.46)	1.04 (0.73, 1.48)	1.03 (0.72, 1.47)
early stage puberty (n=316)	125 (39.6)	1.05 (0.74, 1.48)	1.03 (0.73, 1.46)	1.04 (0.73, 1.47)
prepubescent (n=93)	36 (38.7)	1.01 (0.62, 1.65)	1.01 (0.62, 1.66)	1.06 (0.65, 1.76)
test of association		*P*>0.9	*P*>0.9	*P*>0.9

Analytic samples consist of those with maximum data at each age. Model 1: unadjusted. Model 2: adjusted for birth weight, birth order and childhood illness. Model 3: as for model 2 plus adjustment for father's occupational class. P-values from test of difference between pubertal groups. *Proportions indicate those taking part in LTPA (at least once per month) at each age.

For peer review only - http://bmjopen.bmj.com/site/about/guidelines.xhtml

Table 3 Associations between Age at Menarche and Leisure-Time Physical Activity (LTPA) at each Adult Age in Women from the MRC NSHD, 1946-2014.

	N (%) LTPA*	Odds ratios (95% confidence intervals) of LTPA at least once per month at each adult age versus no LTPA		
		Model 1	Model 2	Model 3
LTPA age 36 years				
≤ 11 (n=216)	118 (54.6)	1.00	1.00	1.00
12 (n=363)	202 (55.6)	0.96 (0.63, 1.45)	0.96 (0.63, 1.45)	1.19 (0.87, 1.62)
13 (n=464)	276 (59.5)	1.14 (0.76, 1.69)	1.14 (0.76, 1.70)	1.05 (0.74, 1.50)
≥ 14 (n=240)	134 (55.8)	1.06 (0.67, 1.67)	1.07 (0.67, 1.70)	1.68 (0.88, 3.24)
test of association		*P*=0.8	*P*=0.8	*P*=0.8
LTPA age 43 years				
≤ 11 (n=208)	82 (39.4)	1.00	1.00	1.00
12 (n=355)	157 (44.2)	1.16 (0.77, 1.74)	1.17 (0.78, 1.77)	1.27 (0.84, 1.93)
13 (n=440)	191 (43.4)	1.21 (0.82, 1.78)	1.24 (0.84, 1.84)	1.31 (0.88, 1.96)
≥ 14 (n=248)	115 (46.4)	1.24 (0.80, 1.92)	1.29 (0.82, 2.02)	1.35 (0.86, 2.13)
test of association		*P*=0.8	*P*=0.7	*P*=0.5
LTPA age 53 years				
≤ 11 (n=203)	102 (50.3)	1.00	1.00	1.00
12 (n=332)	162 (48.8)	0.91 (0.61, 1.37)	0.91 (0.61, 1.38)	0.99 (0.65, 1.50)
13 (n=406)	189 (46.6)	0.82 (0.56, 1.21)	0.84 (0.57, 1.25)	0.90 (0.60, 1.34)
≥ 14 (n=239)	120 (50.2)	1.03 (0.66, 1.59)	1.05 (0.68, 1.64)	1.10 (0.70, 1.72)
test of association		*P*=0.6	*P*=0.7	*P*=0.8
LTPA age 60-64 years				
≤ 11 (n=148)	52 (35.1)	1.00	1.00	1.00
12 (n=270)	101 (37.4)	1.06 (0.68, 1.66)	1.09 (0.69, 1.72)	1.17 (0.74, 1.86)
13 (n=315)	120 (38.1)	1.24 (0.81, 1.92)	1.31 (0.85, 2.04)	1.39 (0.89, 2.17)
≥ 14 (n=169)	62 (36.7)	1.01 (0.62, 1.66)	1.06 (0.64, 1.75)	1.09 (0.66, 1.81)
test of association		*P*=0.7	*P*=0.6	*P*=0.5
LTPA age 68 years				
≤ 11 (n=162)	66 (40.7)	1.00	1.00	1.00
12 (n=280)	108 (38.6)	0.91 (0.62, 1.36)	0.92 (0.62, 1.38)	1.00 (0.66, 1.50)
13 (n=357)	147 (41.2)	1.02 (0.70, 1.49)	1.01 (0.69, 1.48)	1.08 (0.73, 1.59)
≥ 14 (n=186)	80 (43.0)	1.10 (0.72, 1.68)	1.08 (0.70, 1.66)	1.13 (0.73, 1.76)
test of association		*P*=0.8	*P*=0.9	*P*=0.9

Analytic samples consist of those with maximum data at each age. Model 1: unadjusted. Model 2: adjusted for birth weight, birth order and childhood illness. Model 3: as for model 2 plus adjustment for father's occupational class. P-values from test of difference between ages at menarche groups. *Proportions indicate those taking part in LTPA (at least once per month) at each age.

For peer review only - http://bmjopen.bmj.com/site/about/guidelines.xhtml

Supplementary table 1 Associations between Pubertal Status at Age 15 Years and Leisure-Time Physical Activity (LTPA) at each Adult Age in Men from the MRC NSHD after further adjustment for adult physical health, 1946-2014.

	N (%) LTPA*	Odds ratios (95% confidence intervals) of LTPA at least once per month at each adult age versus no LTPA	
		Model A	Model B
LTPA age 36 years			
fully mature (n=316)	230 (72.8)	1.00	1.00
advanced stage puberty (n=395)	258 (65.3)	0.71 (0.51, 0.98)	0.69 (0.50, 0.96)
early stage puberty (n=455)	308 (67.7)	0.78 (0.57, 1.08)	0.76 (0.55, 1.05)
prepubescent (n=131)	97 (74.1)	1.12 (0.70, 1.78)	1.09 (0.69, 1.75)
test of association		P=0.07	P=0.06
LTPA age 43 years			
fully mature (n=293)	169 (57.7)	1.00	1.00
advanced stage puberty (n=363)	176 (48.5)	0.69 (0.50, 0.95)	0.68 (0.49, 0.94)
early stage puberty (n=408)	201 (49.3)	0.74 (0.54, 1.00)	0.71 (0.52, 0.98)
prepubescent (n=119)	58 (48.7)	0.78 (0.50, 1.21)	0.75 (0.48, 1.18)
test of association		P=0.1	P=0.1
LTPA age 53 years			
fully mature (n=258)	135 (52.3)	1.00	1.00
advanced stage puberty (n=314)	164 (52.2)	1.03 (0.73, 1.44)	1.01 (0.72, 1.42)
early stage puberty (n=367)	191 (52.0)	1.03 (0.74, 1.42)	0.98 (0.70, 1.36)
prepubescent (n=106)	54 (51.0)	1.05 (0.66, 1.67)	1.04 (0.65, 1.66)
test of association		P>0.9	P>0.9
LTPA age 60-64 years			
fully mature (n=195)	66 (33.9)	1.00	1.00
advanced stage puberty (n=235)	85 (36.2)	1.15 (0.76, 1.72)	1.13 (0.75, 1.71)
early stage puberty (n=272)	85 (31.3)	0.92 (0.61, 1.37)	0.87 (0.58, 1.31)
prepubescent (n=82)	23 (28.1)	0.84 (0.47, 1.51)	0.84 (0.46, 1.50)
test of association		P=0.6	P=0.5
LTPA age 68 years			
fully mature (n=203)	80 (39.4)	1.00	1.00
advanced stage puberty (n=253)	98 (38.7)	0.97 (0.66, 1.42)	0.96 (0.66, 1.42)
early stage puberty (n=289)	114 (39.5)	0.98 (0.68, 1.43)	0.97 (0.67, 1.41)
prepubescent (n=84)	31 (36.9)	0.94 (0.55, 1.60)	0.94 (0.55, 1.60)
test of association		P>0.9	P>0.9

Analytic samples consist of those with maximum data at each age. Model A: adjusted for birth weight, birth order, childhood illness and father's occupational class. Model B: further adjusted for adult physical health. P-values from test of difference between pubertal groups.

For peer review only

Supplementary table 2 Associations between Age at Menarche and Leisure-Time Physical Activity (LTPA) at each Adult Age in Women from the MRC NSHD after further adjustment for adult physical health, 1946-2014.

	N (%) LTPA*	Odds ratios (95% confidence intervals) of LTPA at least once per month at each adult age versus no LTPA	
		Model A	Model B
LTPA age 36 years			
≤ 11 (n=210)	115 (54.8)	1.00	1.00
12 (n=359)	200 (55.7)	1.07 (0.75, 1.52)	1.03 (0.72, 1.47)
13 (n=457)	270 (59.1)	1.22 (0.87, 1.72)	1.18 (0.84, 1.67)
≥ 14 (n=235)	131 (55.7)	1.08 (0.73, 1.58)	1.00 (0.67, 1.47)
test of association		*P*=0.6	*P*=0.6
LTPA age 43 years			
≤ 11 (n=190)	74 (39.0)	1.00	1.00
12 (n=333)	143 (42.9)	1.25 (0.85, 1.79)	1.22 (0.84, 1.77)
13 (n=407)	173 (42.5)	1.22 (0.85, 1.76)	1.19 (0.83, 1.71)
≥ 14 (n=219)	99 (45.2)	1.36 (0.91, 2.04)	1.30 (0.86, 1.97)
test of association		*P*=0.5	*P*=0.6
LTPA age 53 years			
≤ 11 (n=184)	92 (50.0)	1.00	1.00
12 (n=305)	148 (48.5)	1.01 (0.69, 1.47)	0.99 (0.68, 1.45)
13 (n=377)	170 (45.1)	0.89 (0.61, 1.28)	0.87 (0.60, 1.26)
≥ 14 (n=213)	109 (51.2)	1.13 (0.75, 1.70)	1.08 (0.71, 1.63)
test of association		*P*=0.6	*P*=0.6
LTPA age 60-64 years			
≤ 11 (n=133)	44 (33.1)	1.00	1.00
12 (n=248)	95 (38.3)	1.50 (0.94, 2.39)	1.44 (0.90, 2.30)
13 (n=290)	112 (38.6)	1.56 (0.99, 2.45)	1.54 (0.97, 2.42)
≥ 14 (n=157)	56 (35.7)	1.26 (0.75, 2.09)	1.15 (0.69, 1.93)
test of association		*P*=0.2	*P*=0.2
LTPA age 68 years			
≤ 11 (n=142)	55 (38.7)	1.00	1.00
12 (n=250)	96 (38.4)	1.06 (0.68, 1.63)	1.02 (0.66, 1.57)
13 (n=323)	133 (41.2)	1.15 (0.76, 1.74)	1.13 (0.75, 1.72)
≥ 14 (n=164)	66 (40.2)	1.11 (0.69, 1.78)	1.03 (0.64, 1.66)
test of association		*P*>0.9	*P*=0.9

Analytic samples consist of those with maximum data at each age. Model A: adjusted for birth weight, birth order, childhood illness and father's occupational class. Model B: further adjusted for adult physical health. P-values from test of difference between pubertal groups.

For peer review only

STROBE Guidelines for authors of CORR

To be used by authors of all observational clinical studies published in CORR. For this purpose a cohort study (the term used by STROBE) is considered a longitudinal study typically reporting outcomes of treatment in one or more cohorts; a case-control study is one identifying factors in outcomes; a cross-sectional study is one to identify the prevalence of factors or characteristics in a population at a single point in time.

This table is modified from and used with the permission of The STROBE Initiative, www.strobe-statement.org.

Modifications: We added a fourth column for authors to check inclusion. You must include all items in your manuscript unless the information is not applicable. Information on the study cohort (Items 13 and 14 in the STROBE guidelines) should be provided in Patients and Methods, not in Results; we have omitted the portions of the STROBE guidelines related to Results and Discussion (see our guidelines). The STROBE guidelines were developed for epidemiological studies; “exposed” or “exposure” have been modified with the words “treated” or “treatment.”

STROBE Statement—Checklist of items that should be included in reports of ***case-control studies***

	**Item No**	**Recommendation**	**Please insert check where included or N/A where not applicable**
Title and abstract	1	(*a*) Indicate the study’s design with a commonly used term in the title or the abstract	1-2
		(*b*) Provide in the abstract an informative and balanced summary of what was done and what was found	2-3
		**Introduction**	
Background/rationale	2	Explain the scientific background and rationale for the investigation being reported	4-5
Objectives	3	State specific objectives, including any prespecified hypotheses	5
		**Methods**	
Study design	4	Present key elements of study design early in the paper	6-8
Setting	5	Describe the setting, locations, and relevant dates, including periods of recruitment, treatment, follow-up, and data collection	6
Participants	6	(*a*) *Cohort study*—Give the eligibility criteria, and the sources and methods of selection of participants. Describe methods of follow-up *Case-control study*—Give the eligibility criteria, and the sources and methods of case ascertainment and control selection. Give the rationale for the choice of cases and controls	6

For peer review only - http://bmjopen.bmj.com/site/about/guidelines.xhtml

Cross-sectional study—Give the eligibility criteria, and the sources and methods of selection of participants	
(*b*) *Cohort study*—For matched studies, give matching criteria and number of treated and untreated<br>*Case-control study*—For matched studies, give matching criteria and the number of controls per case	NA

For peer review only

For peer review only - http://bmjopen.bmj.com/site/about/guidelines.xhtml

Participants	13*	(a) Report numbers of individuals at each stage of study—eg numbers potentially eligible, examined for eligibility, confirmed eligible, included in the study, completing follow-up, and analysed	6
		(b) Give reasons for non-participation at each stage	6-7
Descriptive data	14*	(a) Give characteristics of study participants (eg demographic, clinical, social) and information on other treatments and potential confounders	10, table 1
		(b) Indicate number of participants with missing data for each variable of interest	6-7, 10
		(c) *Cohort study*—Summarise follow-up time (eg, average and total amount)	6-7
Variables	7	Clearly define all outcomes, treatments, predictors, potential confounders, and effect modifiers. Give diagnostic criteria, if applicable	7-9
Data sources/ measurement	8*	For each variable of interest, give sources of data and details of methods of assessment (measurement). Describe comparability of assessment methods if there is more than one group	6-9
Bias	9	Describe any efforts to address potential sources of bias	7-9, 13-14
Study size	10	Explain how the study size was arrived at	6-7
Quantitative variables	11	Explain how quantitative variables were handled in the analyses. If applicable, describe which groupings were chosen and why	7-8
Statistical methods	12	(*a*) Describe all statistical methods, including those used to control for confounding	8-9
		(*b*) Describe any methods used to examine subgroups and interactions	8-9
		(*c*) Explain how missing data were addressed	7-10
		(*d*) *Cohort study*—If applicable, explain how loss to follow-up was addressed<br>*Case-control study*—If applicable, explain how matching of cases and controls was addressed<br>*Cross-sectional study*—If applicable, describe analytical methods taking account of sampling strategy	6-10
		(*e*) Describe any sensitivity analyses	7-9, 14

*Give information separately for cases and controls.

Note: An Explanation and Elaboration article discusses each checklist item and gives methodological background and published examples of transparent reporting. The STROBE checklist is best used in conjunction with this article (freely available on the Web sites of PLoS Medicine at http://www.plosmedicine.org/, Annals of Internal Medicine at http://www.annals.org/, and Epidemiology at http://www.epidem.com/). Information on the STROBE Initiative is available at http://www.strobe-statement.org.

For peer review only - http://bmjopen.bmj.com/site/about/guidelines.xhtml

BMJ Open

Markers of pubertal timing and leisure-time physical activity from ages 36 to 68 years: findings from a British birth cohort

Journal:	*BMJ Open*
Manuscript ID	bmjopen-2017-017407.R2
Article Type:	Research
Date Submitted by the Author:	13-Sep-2017
Complete List of Authors:	Elhakeem, Ahmed; University of Bristol School of Clinical Science,<br>Cooper, Rachel; UCL, MRC Unit for Lifelong Health and Ageing at UCL<br>Bann, David; University College London, UCL Institute of Education<br>Kuh, Diana; MRC Unit for Lifelong Health and Ageing at UCL,<br>Hardy, Rebecca; University College London, MRC Unit for Lifelong Health and Ageing
**Primary Subject Heading**:	Epidemiology
Secondary Subject Heading:	Public health
Keywords:	Life course, leisure-time, physical activity, pubertal timing

SCHOLARONE™ Manuscripts

For peer review only

Markers of pubertal timing and leisure-time physical activity from ages 36 to 68 years: findings from a British birth cohort

Ahmed Elhakeem, PhD[1,2]*, Rachel Cooper, PhD[1], David Bann, PhD[3], Diana Kuh, PhD[1], Rebecca Hardy, PhD[1]

[1]MRC Unit for Lifelong Health and Ageing at UCL, London, UK

[2]Musculoskeletal Research Unit, School of Clinical Sciences, University of Bristol, Bristol, UK

[3] Centre for Longitudinal Studies, UCL Institute of Education, London, UK

*Correspondence: Musculoskeletal Research Unit, School of Clinical Sciences, University of Bristol, Level 1 Learning and Research Building, Southmead hospital, Bristol BS10 5NB, UK, Email: a.elhakeem@bristol.ac.uk, Tel: 0044(0)1174147864

Abstract = 300 words, Text = 3686 words, 40 References, 3 Tables (2 Supplementary files)

For peer review only

ABSTRACT

Objectives

We aimed to examine associations between markers of pubertal timing and leisure-time physical activity (LTPA) from ages 36 to 68 years in men and women from the MRC National Survey of Health and Development.

Study design

Pubertal timing was ascertained by physicians at age 14-15. Boys were grouped, based on their secondary sexual characteristics, as prepubescent, in early stage puberty, advanced stage puberty or fully mature at age 14-15. Girls were grouped as reaching menarche ≤11, 12, 13 or ≥14 years. LTPA was reported at ages 36, 43, 53, 60-64 and 68 and classified as active or inactive at each age. Associations were examined using standard and mixed-effects logistic regression models.

Results

Of 5,362 singleton births recruited, 1499 men and 1409 women had at least one measure of LTPA and data on pubertal timing and selected covariates. When compared with men that were fully mature at age 14-15, those that were in advanced and early stage puberty, but not the prepubescent stage, had lower likelihood of LTPA at younger but not older adult ages (P=0.06 for pubertal status-by-age at LTPA interaction in mixed-effects model). For example, fully-adjusted odds ratios (OR) of LTPA (versus no LTPA) at ages 36 and 68 years respectively for advanced puberty versus fully mature were 0.69 (95% confidence intervals: 0.50, 0.96) and 1.03 (0.72, 1.47). Age at menarche was not associated with LTPA at any age ($P_{\text{interaction}}$ with age at LTPA=0.9). For example, OR (from mixed-effects model) of LTPA between 36 and 68 years was 1.23 (0.93, 1.63) for menarche at 13 versus ≤11 years.

For peer review only - http://bmjopen.bmj.com/site/about/guidelines.xhtml

Conclusions

In a nationally representative study, there was little evidence to suggest that pubertal timing was an important correlate of LTPA between ages 36 and 68 years. Maturity-related variations in adolescents' LTPA may be transitory and lose importance over time.

KEY WORDS

Life course; leisure-time; physical activity; pubertal timing.

STRENGTHS AND LIMITATIONS OF THIS STUDY

- Assessment of boys' pubertal status at age 14-15 and girls' age at menarche by physicians at school-based medical exams.
- Leisure-time physical activity repeatedly measured prospectively throughout adulthood.
- Limited by fewer numbers of participants at the extremes of pubertal age and lack of information on physical activity participation in childhood and prior to age 36.
- Birth cohort effects and contextual differences in what leisure-time physical activity signifies in adolescence and adulthood may contribute to findings.

For peer review only - http://bmjopen.bmj.com/site/about/guidelines.xhtml

BACKGROUND

Leisure-time physical activity (LTPA) promotes health and therefore it is important to identify groups that might benefit from support to participate in LTPA. Adolescence is a key developmental stage where health behaviours, such as LTPA habits are initiated (1, 2), and which can track into adulthood (3). A major feature of the adolescent period is puberty; a complex process spanning several related domains of growth and development through which adolescents develops into sexually mature adults (4). Besides the importance of adolescence and puberty for the development of health behaviours, deviation from the normal timing of the pubertal process might also influence LTPA (5, 6). Two hypotheses are thought to support this argument; the early maturation hypothesis, which proposes that early maturing adolescents, due to interruptions to their normal course of behavioural development, are at risk of adopting unhealthy behaviour (7), and the maturation deviance hypothesis which suggests that both early and late onset of puberty are associated with subsequent psychosocial problems (8).

Systematic reviews, of mostly cross-sectional studies, suggest that early maturing girls tend to participate less in sports activities than girls who reach their marker of puberty around the average age, thus supporting the early maturation hypothesis (5, 6). Conversely, in males, who are less frequently studied, late maturing boys are generally found to participate less in sports activities than boys reaching their marker of puberty around the average age, favouring the maturation deviance hypothesis in relation to negative health outcomes for later maturing boys (5, 6). While underlying processes are likely complex, it is thought that greater psychosocial problems such as more negative perceptions of their physical appearance and less enjoyment of sports by earlier maturing girls may discourage their participation in sports (5, 6, 9, 10). On the other hand, greater physical strength and perceived competence at sports among earlier maturing boys may encourage their participation in sports (5, 6, 9-12).

For peer review only - http://bmjopen.bmj.com/site/about/guidelines.xhtml

However, it is unclear if maturity-related differences in adolescents LTPA are observed in adults.

Both earlier and later timing of puberty have also been associated with health outcomes in later life that could influence participation in LTPA in adult life. For example, findings from UK Biobank showed both earlier and later ages at puberty were associated with a range of chronic disease outcomes, including heart disease and psychiatric disorders, in both men and women (13). In the MRC National Survey of Health and Development (NSHD), earlier puberty was associated in midlife with greater body mass index (14), higher levels of triglycerides in women (15) and higher blood pressure (14), and greater android fat mass in men (16), whereas later puberty was associated with less bone mineral density (17).

To our knowledge, only three studies have examined associations of age at puberty with LTPA in adults. Perceived relative pubertal status reported by 12-19-year-old Norwegians was not related to LTPA over thirteen years of follow-up in one study (18), and in the 1958 National Child Development Study (NCDS), age at puberty was unrelated to midlife LTPA (19). However, among 166 40-year old Belgian men (20), later age at peak height velocity (APHV), a marker of later pubertal onset, correlated with higher participation in sports. It is possible that associations between pubertal timing and LTPA weaken with age, as the pathways on which puberty acts become less important influences on LTPA, or possibly due to health conditions associated with early or late puberty being associated with declines or improvement in health and LTPA. Therefore, detailed studies with repeated measures of LTPA across adult life are needed, including those that examine associations in both male and female samples. This study examines associations of age at menarche in women and pubertal stage at age 14-15 in men with LTPA from ages 36 to 68 years, and whether any associations found change with age at assessment of LTPA.

For peer review only - http://bmjopen.bmj.com/site/about/guidelines.xhtml

METHODS

Participants

The NSHD is a nationally representative sample of 5,362 British singleton births during one week in March 1946 and followed up regularly across childhood and adulthood (21, 22). In childhood and adolescence, data on NSHD participants were collected from parents, health care and educational professionals and study participants themselves. At age 14-15 (1961), medical examinations were carried out by school doctors. At ages 36 (1982), 43 (1989) and 53 (1999) trained nurses interviewed and assessed the study participants in their own homes. At age 60-64 (2006-2010) they attended one of 6 clinical research facilities or received a home visit, and at age 68 (2014) they completed a postal questionnaire followed by a home visit.

Of those successfully contacted at ages 36 (n=3322), 43 (n=3262), 53 (n=3035), 60-64 (n=2661) and 68 years (n=2453), 99.6%, 100%, 98.4%, 82.2% and 99.1% respectively provided information on LTPA (3766 participants had at least one measure of LTPA). The participating samples at ages 43, 53 and 60-64 have been found to be broadly representative of similar aged members of the general UK population (23-25). At the most recent round of data collection at age 68, of the 2816 people in the target sample living in England, Scotland and Wales, 2370 (84.2%) completed a postal questionnaire. In addition, a postal questionnaire was sent to 126 study members living abroad who remain in contact with the study of whom 83 (65.9%) returned a questionnaire. No attempt was made to contact the remaining 2420 study members: 957 (17.8%) had already died, 620 (11.6%) had previously withdrawn from the study, 448 (8.3%) had emigrated and were no longer in contact with the study, and 395 (7.4%) had been untraceable for more than five years (26).

For peer review only - http://bmjopen.bmj.com/site/about/guidelines.xhtml

Relevant ethics approval has been granted for each data collection; ethical approval for the most recent assessment in 2014 was obtained from the Queen Square Research Ethics Committee (14/LO/1073) and the Scotland A Research Ethics Committee (14/SS/1009). Study participants provided written informed consent.

Measurements

Pubertal measurements

In 1961, when study members were aged 14-15 (mean age 14.5), they (both boys and girls) were assessed as part of a school-based medical exam carried out in school clinics by physicians with a parent present (14, 15, 17, 24). These physical examinations, which predated Tanner staging, included assessments of the visibility of pigmented pubic hair (none, sparse, profuse), visibility of axillary hair (no, yes) and, for boys only, development of genitalia (infantile, early, advanced), and whether the voice had broken (no, starting, completely broken). On the basis of these four indicators of secondary sexual characteristics, boys were grouped as prepubescent (infantile genitalia or early adolescent genitalia but no pubic or axillary hair and voice not broken), early stage puberty (early development of genitalia and some pubic or axillary hair or voice starting to break), advanced stage puberty (advanced development of genitalia but at least one other indicator not fully mature) or fully mature (advanced development of genitalia, and profuse pubic hair and axillary hair and voice broken) at age 14-15.

Mothers reported their daughters' age at menarche at the age 14-15 school-based medical exam. In addition to a continuous measure of age at menarche, women were also grouped as reaching menarche ≤11 years, 12 years, 13 years or ≥14 years, similar to a previous study examining the association with LTPA (19). All women were asked to recall their age at menarche at age 48. Women that had not reached menarche by the age 14-15 medical exam

For peer review only

and who subsequently reported reaching menarche at an age that was consistent with this (i.e. at/after age 14-15) were included (in the ≥14 years group). Recalled age at menarche was not used to replace values if they were missing from mother's report due to evidence that recall of age at menarche may not be valid (27). For these analyses, we use pubertal status at age 14-15 in males and age at menarche in females as markers of pubertal timing (4).

Leisure-time physical activity from ages 36 to 68 years

At ages 36, 43, 53, 60-64 and 68, study participants reported how often they participated in LTPA during nurse interviews or using self-completed questionnaires (6). At age 36, study participants reported the number of times they took part in 27 different sports, exercises and other leisure activities during the previous month using questions based on the Minnesota LTPA questionnaire (7). At age 43, information was collected on participation in sports, exercise or other vigorous leisure activities in the previous year including for how many months and how often in those months activities were performed (6, 8). At ages 53, 60-64 and 68, study participants were asked how often they participated in sports, exercise or other vigorous leisure activities during the previous four weeks. At each age, study participants were classed as active if they reported taking part at least once in LTPA (in the previous month at age 36, per month at age 43, and in the previous 4 weeks at ages 53, 60-64 and 68) or inactive (no LTPA).

Potential confounders

Potential confounders were selected *a priori.* These were birth weight, birth order, childhood illness and father's occupational class, all of which had been identified from the literature as being associated with both pubertal timing and LTPA (28-32). Birth weights were extracted from birth records and information was obtained from mothers on birth order and serious illness in the first 5 years of life (33, 34). Father's Registrar General's occupational class at

For peer review only - http://bmjopen.bmj.com/site/about/guidelines.xhtml

age 4 (in 1950) (or at age 11 or 15 if missing at age 4 (n=173)) was used to indicate childhood socioeconomic position and was grouped into four categories (I&II: professional, managerial or technical, IIINM: skilled non-manual, IIIM: skilled manual and IV&V: partly skilled or unskilled).

Statistical analyses

Models were fit separately for men and women due to differences in the way pubertal timing had been assessed, and expected differences in patterns of association with LTPA (5, 6). Associations between women's age at menarche and men's pubertal stage at 14-15 and LTPA were examined using standard logistic regression models for LTPA at each age from 36 to 68 years. Initial (unadjusted) models (Model 1) were adjusted for birth weight, birth order and childhood illness (Model 2) and additionally for father's occupational class in final models (Model 3).

To examine whether any associations were mediated by health in adulthood, final models were refitted with additional adjustment for physical health status at age 36. Physical health was derived from information collected during nurse interviews at age 36 years on weight, disability, self-reported health problems, hypertension, lung function and incidence of hospital admissions (35). This information was used to categorise participants into worst, intermediate or best physical health (35). To formally test whether the association between pubertal timing and probability of LTPA changes with increasing age we combined all participants with at least one measure of LTPA and fit mixed-effects logistic regression models (25) containing pubertal timing-by-age at LTPA interaction terms. These models were adjusted for all selected confounders and fitted with random intercepts and slopes for age at assessment of LTPA and an independent correlation structure. An assumption of

For peer review only - http://bmjopen.bmj.com/site/about/guidelines.xhtml

linearity was used for examining whether associations change with age at assessment of LTPA. All analyses were carried out in STATA 14 (StataCorp, College Station, TX).

RESULTS

Participant characteristics

In total, 1499 men and 1409 women had at least one measure of adulthood LTPA and complete data on age at puberty (pubertal status/menarche) and all selected covariates. A majority of included participants had LTPA data from four of the five different ages in adulthood (a total of 5621 and 5601 LTPA assessments were included in men and women analyses respectively). When compared with those excluded due to missing LTPA data (n=1,596), higher proportions of those with at least one measure of LTPA were female (49.6% vs. 42.5%) and had fathers in occupational classes I & II (23.1% v 20.9%) and lower proportions had low birth weight (4.7% vs. 8.8%) and serious childhood illness (6.5% vs. 17.7%). Over 80% of women had reached menarche by ages 13 years and a quarter of men were fully mature at age 15 (Table 1). Higher proportions of men reported taking part in LTPA at ages 36 and 43 but sex-differences were less marked at older ages (Table 1).

Men: pubertal status at age 15 and LTPA from 36 to 68 years

Weak associations were found suggesting that the earliest maturing boys (i.e. those classified as fully mature at age 15) were more likely to participate in LTPA at ages 36 and 43, when compared with boys in advanced and early stage puberty at age 15. At age 43 but not 36, there was also a suggestion that the latest maturing boys were less likely than the earliest maturing boys to participate in LTPA (Table 2). No such associations were observed with LTPA at older ages. Adjustment for birth weight, birth order, childhood illness and father's occupational class had little influence on estimates (Table 2). Further adjustment for physical health status at age 36 had little influence on estimates (supplementary table 1).

For peer review only - http://bmjopen.bmj.com/site/about/guidelines.xhtml

The finding of stronger associations at ages 36 and 43 is supported by evidence that associations changed with age at assessment of LTPA when formally tested using mixed-effects models (*P*=0.06 for boys' pubertal status-by-age at LTPA interaction).

Women: age at menarche and LTPA from 36 to 68 years

There was no evidence that age at menarche was associated with LTPA between 36 to 68 years (Table 3). There was also no evidence of an interaction between age at menarche and age at assessment of LTPA when tested using the mixed-effect models (*P*=0.9 for categorical and continuous age at menarche-by-age at LTPA interactions) suggesting that findings did not differ by the age at assessment of LTPA. The fully-adjusted ORs of LTPA between 36 and 68 years (estimated with mixed-effects models) for those reaching menarche at 12, 13 and ≥14 years (versus 11 years) were 1.14 (0.85, 1.52), 1.23 (0.93, 1.63) and 1.18 (0.86, 1.63) respectively (*P*=0.6 for difference between menarche groups). Further adjustment for physical health status at age 36 did not influence the estimated ORs of LTPA at each age (supplementary table 2).

DISCUSSION

Main findings

This study examined whether markers of pubertal timing in males and females (clinical assessments of men's pubertal status based on developmental stages of secondary sexual characteristics at age 14-15 and prospective reporting of women's age at menarche) were related to LTPA from ages 36 to 68 years in the MRC NSHD (1946 British birth cohort). There was weak evidence that, when compared with the earliest maturing boys (i.e. fully mature at age 14-15), those in advanced and early stages of puberty, but not the prepubescent stage, were less likely to participate in LTPA at ages 36 and 43, and that differences in LTPA

For peer review only - http://bmjopen.bmj.com/site/about/guidelines.xhtml

by pubertal status attenuated with increasing age. Age at menarche was not associated with adulthood LTPA.

Comparison with other studies

Very few studies have examined how markers of pubertal timing relate to LTPA in adulthood. Largely consistent with our findings are results from the next oldest British birth cohort (1958 NCDS) where the authors reported no associations between stages of boys' axillary hair development and menarche age in girls and LTPA between ages 33-50 years but did not present estimates or test whether associations changed with age (19). Our findings are also largely consistent with null associations reported between self-rated maturity status reported by 12-19 year-old Swedes and subsequent LTPA assessed three times over thirteen years follow-up (18).

In keeping with our findings, Wichstrøm et al. (18) found weak associations between earlier pubertal timing and more LTPA but only at the youngest (mean age=16.5 years) and not the two older follow-up ages to early adulthood (mean ages 21.5 and 28.5 respectively) however, both males and females were included in their analyses so it is unclear if their findings varied by sex (18). In contrast to our finding that early maturing boys appeared more likely to participate in LTPA at ages 36 and 43, Beunen and colleagues (2004) found that later maturity as indicated by later APHV correlated with more participation in sports in 40-year old Belgian men however, APHV was not associated with a leisure-time activities index that excluded sports (20). Finally, that early maturing boys appeared more likely to participate in LTPA at younger adult ages in the NSHD is consistent with findings from adolescent studies that show early maturing boys tend to be more active in sports than their later maturing peers (5, 6).

Explanation of findings

For peer review only - http://bmjopen.bmj.com/site/about/guidelines.xhtml

For peer review only

Overall, our findings suggest that pubertal timing is not a sizeable correlate of LTPA across adulthood (from 36 to 68 years) in either men or women. One explanation is that maturity-related variations in the LTPA of adolescents (5, 6, 11) may represent transitionary effects on PA which diminish in importance once all peers move beyond puberty into adulthood. Consistent with this, it could be that the differences in LTPA observed in this study between boys in different pubertal groups at age 15 declined with age because some men who were very active as adolescents and young adults (e.g. through frequent involvement in team sports) reduced their participation in team sports as they get older. This is supported by studies which found late adolescence as a period when many drop out of team sports (36, 37) and findings that chronological age was a more important predictor of PA in older adolescents than pubertal timing (38). Other possible explanations for our findings could include birth cohort differences in associations of pubertal timing with later LTPA, and differences in what LTPA captures in adolescence and adulthood.

Methodological considerations

This study benefits from an investigation of age-related changes in associations of pubertal timing with LTPA, where we included all individuals with at least one measure of LTPA under the missing at random assumption, examining association separately in males and females and adjustment for prospectively ascertained covariates. In addition, loss to follow-up in NSHD led to only slight differences in characteristics between those included and those with missing data. The prospective ascertainment by school-based physicians of men's pubertal status at age 14-15 and women's age at menarche is another notable strength.

Limitations include relatively small numbers of participants in the extreme puberty groups (e.g. <11 for age at menarche in girls) which would have reduced statistical power. This meant it was necessary to combine all those girls who reached menarche by 11 years into a

For peer review only - http://bmjopen.bmj.com/site/about/guidelines.xhtml

single group (though this is similar to the cut-point used for early menarche in the large UK Biobank study of pubertal timing and adult disease outcomes (9)) and those reaching menarche at or after 14 years into a single group. Since studies show that the extremes of early and late pubertal timing are those at risk of adverse health outcomes (13), it may be that it is those very extreme groups where differences in LTPA are observed and which could not be tested in NSHD due to lower statistical power. However, further investigation revealed no evidence of quadratic effects of age at menarche (P=0.8 for quadratic menarche term from mixed-effects model), and further, parametrising continuous age at menarche using mixed-effects fractional polynomial models also led to similar null findings (all P-values from partial F tests ≥0.9).

While the markers of pubertal timing used here were prospectively assessed, some misclassification in the categories used may still be possible. In addition, we speculated that adult health may mediate associations between puberty timing and LTPA however, it is likely that there is a bidirectional process such that LTPA also mediates associations between pubertal timing and adult health. Further, the markers of pubertal timing used were not directly comparable for males and females, and the historical nature of these assessments meant we did not have data on other markers of puberty such as Tanner staging.

LTPA was self-reported therefore, recall and misclassification errors are possible however, self-reports are required to capture activity within specific domains like LTPA (39). Further, in a subsample of this cohort, those participating in LTPA across adulthood were found to spend greater time in moderate-to-vigorous intensity PA as assessed by activity monitors when compared with others reporting no LTPA (40). In addition, our classification of LTPA as at least once per month at each age, which is similar to how we dichotomised LTPA in our recent study (25), may be a limitation given it summarised activity according to whether or not any participation in LTPA was reported, however, findings were similar when using a

For peer review only - http://bmjopen.bmj.com/site/about/guidelines.xhtml

higher LTPA cut-off of at least five times a month (versus up to 4 times per month) at each adult age. Further, that the LTPA measures were dichotomous in nature limits the amount of LTPA information used however, this does provide comparable measures to allow tracking of LTPA over 32 years. Additionally, we only examined LTPA in this study and other activity domains (e.g. active transport) may be important for overall PA levels. Furthermore, while we adjusted for several early life confounders, we did not have data on early life LTPA which may be important given the tracking of LTPA across life, and it is possible that maturity-related variations in LTPA may be less pronounced in those with a high level of sports during childhood (6). Finally, residual confounding due to unmeasured confounders and measurement error in the measured confounders could influence findings.

Conclusions

In a nationally representative prospective British birth cohort study, pubertal timing, based on clinical assessments of stages of secondary sexual characteristics at age 14-15 in boys and the age at menarche in girls, did not appear to be an important correlate of LTPA reported from ages 36 to 68 years. Age at menarche was not associated with LTPA whereas weak evidence was found that, when compared with the earliest maturing boys, those in advanced and early stages of puberty, but not prepubescent stage, were less likely to participate in LTPA at younger but not older adult ages, and that differences in LTPA by men's pubertal status at age 14-15 attenuated with increasing adult age. Thus, associations between pubertal timing and adolescent LTPA reported in other studies (5, 6, 10, 11, 38), may be transient and lose importance over time and once all peers have transitioned into adulthood.

ACKNOWLEDGMENTS

For peer review only - http://bmjopen.bmj.com/site/about/guidelines.xhtml

We thank the study participants for their continuing participation in the MRC National Survey of Health and Development (NSHD). We also thank members of the NSHD scientific and data collection teams who have been involved in the NSHD data collections.

FUNDING

This work was supported by the UK Medical Research Council [programme codes: MC_UU_12019/1]; the funder had no role in the design of the study or the writing of the manuscript and played no part in the decision to submit it for publication.

CONTRIBUTORSHIP STATEMENT

AE, RC, DB, DK and RH designed the study. AE performed the data analysis and produced the first draft of the manuscript. All authors contributed to the development of the manuscript and read and approved its final version.

COMPETING INTERESTS

The authors declare that they have no competing interests.

DATA SHARING STATEMENT

Data used in this publication are available to bona fide researchers upon request to the NSHD Data Sharing Committee via a standard application procedure. Further details can be found at http://www.nshd.mrc.ac.uk/data. doi: 10.5522/NSHD/Q101; doi: 10.5522/NSHD/Q102; doi: 10.5522/NSHD/Q103.

For peer review only - http://bmjopen.bmj.com/site/about/guidelines.xhtml

REFERENCES

1. Patton GC, Viner R. Pubertal transitions in health. Lancet. 2007;369(9567):1130-9.

2. Schooling CM. Life course epidemiology: recognising the importance of puberty. J Epidemiol Community Health. 2015;69(8):820.

3. Telama R. Tracking of physical activity from childhood to adulthood: a review. Obes Facts. 2009;2(3):187-95.

4. Dorn LD, Dahl RE, Woodward HR, Biro F. Defining the boundaries of early adolescence: a user's guide to assessing pubertal status and pubertal timing in research with adolescents. Appl Dev Sci. 2006;10(1):30-56.

5. Bacil ED, Mazzardo Junior O, Rech CR, Legnani RF, de Campos W. [Physical activity and biological maturation: a systematic review]. Rev Paul Pediatr. 2015;33(1):114-21.

6. Sherar LB, Cumming SP, Eisenmann JC, Baxter-Jones AD, Malina RM. Adolescent biological maturity and physical activity: biology meets behavior. Pediatr Exerc Sci. 2010;22(3):332-49.

7. Petersen A, Taylor B, Aldelson J. The biological approach to adolescence: Biological change and psychosocial adaptation. Handbook of adolescent psychology. New York: Wiley; 1980. p. 117–55.

8. Alasker F. Timing of puberty and reactions to pubertal changes. In: Rutter M, editor. Psychosocial disurbances in young people. Cambridge: Cambridge University Press; 1995. p. 37-82.

9. Lee EY, An K, Jeon JY, Rodgers WM, Harber VJ, Spence JC. Biological maturation and physical activity in South Korean adolescent girls. Med Sci Sports Exerc. 2016;48(12):2454-61.

For peer review only - http://bmjopen.bmj.com/site/about/guidelines.xhtml

10. Davison KK, Werder JL, Trost SG, Baker BL, Birch LL. Why are early maturing girls less active? Links between pubertal development, psychological well-being, and physical activity among girls at ages 11 and 13. Soc Sci Med. 2007;64(12):2391-404.

11. Cumming SP, Standage M, Gillison FB, Dompier TP, Malina RM. Biological maturity status, body size, and exercise behaviour in British youth: a pilot study. J Sports Sci. 2009;27(7):677-86.

12. Mrug S, Elliott MN, Davies S, Tortolero SR, Cuccaro P, Schuster MA. Early puberty, negative peer influence, and problem behaviors in adolescent girls. Pediatrics. 2014;133(1):7-14.

13. Day FR, Elks CE, Murray A, Ong KK, Perry JR. Puberty timing associated with diabetes, cardiovascular disease and also diverse health outcomes in men and women: the UK Biobank study. Sci Rep. 2015;5:11208.

14. Hardy R, Kuh D, Whincup PH, Wadsworth ME. Age at puberty and adult blood pressure and body size in a British birth cohort study. J Hypertens. 2006;24(1):59-66.

15. Pierce MB, Kuh D, Hardy R. Role of lifetime body mass index in the association between age at puberty and adult lipids: findings from men and women in a British birth cohort. Ann Epidemiol. 2010;20(9):676-82.

16. Ong KK, Bann D, Wills AK, Ward K, Adams JE, Hardy R, et al. Timing of voice breaking in males associated with growth and weight gain across the life course. J Clin Endocrinol Metab. 2012;97(8):2844-52.

17. Kuh D, Muthuri SG, Moore A, Cole TJ, Adams JE, Cooper C, et al. Pubertal timing and bone phenotype in early old age: findings from a British birth cohort study. Int J Epidemiol. 2016.

18. Wichstorm L, Kvalem IL, Soest T. Predictors of growth and decline in leisure-time physical activity from adolescence to adulthood. Health Psychol. 2013;32(7):775-84.

For peer review only

For peer review only - http://bmjopen.bmj.com/site/about/guidelines.xhtml

19. Pinto Pereira SM, Li L, Power C. Early-life predictors of leisure-time physical inactivity in midadulthood: findings from a prospective British birth cohort. Am J Epidemiol. 2014;180(11):1098-108.

20. Beunen GP, Lefevre J, Philippaerts RM, Delvaux K, Thomis M, Claessens AL. Adolescent correlates of adult physical activity: a 26-year follow-up. Med Sci Sports Exerc. 2004;36(11):1930-6.

21. Kuh D, Pierce M, Adams J, Deanfield J, Ekelund U, Friberg P, et al. Cohort profile: updating the cohort profile for the MRC National Survey of Health and Development: a new clinic-based data collection for ageing research. Int J Epidemiol. 2011;40(1):e1-9.

22. Wadsworth M, Kuh D, Richards M, Hardy R. Cohort Profile: The 1946 National Birth Cohort (MRC National Survey of Health and Development). Int J Epidemiol. 2006;35(1):49-54.

23. Stafford M, Black S, Shah I, Hardy R, Pierce M, Richards M, et al. Using a birth cohort to study ageing: representativeness and response rates in the National Survey of Health and Development. Eur J Ageing. 2013;10(2):145-57.

24. Wadsworth M, Butterworth SL, Hardy RJ, Kuh DJ, Richards M, Langenberg C, et al. The life course prospective design: an example of benefits and problems associated with study longevity. Soc Sci Med. 2003;57(11):2193-205.

25. Wadsworth M, Mann SL, Rodgers B, Kuh DJ, Hilder WS, Yusuf EJ. Loss and representativeness in a 43 year follow up of a national birth cohort. J Epidemiol Community Health. 1992;46(3):300-4.

26. Kuh D, Wong A, Shah I, Moore A, Popham M, Curran P, et al. The MRC National Survey of Health and Development reaches age 70: maintaining participation at older ages in a birth cohort study. Eur J Epidemiol. 2016;31(11):1135-47.

For peer review only - http://bmjopen.bmj.com/site/about/guidelines.xhtml

27. Cooper R, Blell M, Hardy R, Black S, Pollard TM, Wadsworth ME, et al. Validity of age at menarche self-reported in adulthood. J Epidemiol Community Health. 2006;60(11):993-7.

28. Bauman AE, Reis RS, Sallis JF, Wells JC, Loos RJ, Martin BW. Lancet Physical Activity Series Working Group: correlates of physical activity: why are some people physically active and others not? Lancet. 2012;380(9838):258-71.

29. Elhakeem A, Cooper R, Bann D, Hardy R. Childhood socioeconomic position and adult leisure-time physical activity: a systematic review. Int J Behav Nutr Phys Act. 2015;12(1):1-27.

30. Elhakeem A, Cooper R, Bann D, Kuh D, Hardy R. Birth weight, school sports ability, and adulthood leisure-time physical activity. Med Sci Sports Exerc. 2017;49(1):64-70.

31. Malina RM BC, Bar-Or O. Biological maturation: concepts and assessment. In: Malina RM BC, Bar-Or O, editor. Growth, maturation, and physical activity. United States: Human Kinetics; 2004. p. 277-306.

32. Mishra GD, Cooper R, Tom SE, Kuh D. Early life circumstances and their impact on menarche and menopause. Womens Health (Lond). 2009;5(2):175-90.

33. Kuh DJ, Cooper C. Physical activity at 36 years: patterns and childhood predictors in a longitudinal study. J Epidemiol Community Health. 1992;46(2):114-9.

34. Muthuri SG, Kuh D, Bendayan R, Macfarlane GJ, Cooper R. Chronic physical illness in early life and risk of chronic widespread and regional pain at age 68: evidence from the 1946 British birth cohort. Pain. 2016;157(10):2382-9.

35. Kuh DJ, Wadsworth ME. Physical health status at 36 years in a British national birth cohort. Soc Sci Med. 1993;37(7):905-16.

36. Lunn PD. The sports and exercise life-course: a survival analysis of recall data from Ireland. Soc Sci Med. 2010;70(5):711-9.

For peer review only

For peer review only - http://bmjopen.bmj.com/site/about/guidelines.xhtml

37. Zick CD, Smith KR, Brown BB, Fan JX, Kowaleski-Jones L. Physical activity during the transition from adolescence to adulthood. J Phys Act Health. 2007;4(2):125-37.

38. Finne E, Bucksch J, Lampert T, Kolip P. Age, puberty, body dissatisfaction, and physical activity decline in adolescents. Results of the German Health Interview and Examination Survey (KiGGS). Int J Behav Nutr Phys Act. 2011;8:119.

39. Troiano RP, McClain JJ, Brychta RJ, Chen KY. Evolution of accelerometer methods for physical activity research. Br J Sports Med. 2014;48(13):1019-23.

40. Golubic R, Martin KR, Ekelund U, Hardy R, Kuh D, Wareham N. Levels of physical activity among a nationally representative sample of people in early old age: results of objective and self-reported assessments. Int J Behav Nutr Phys Act. 2014;11.

For peer review only

For peer review only - http://bmjopen.bmj.com/site/about/guidelines.xhtml

Table 1 Descriptive Characteristics by Sex, the Medical Research Council (MRC) National Survey of Health and Development (NSHD), 1946-2014.

	Males (n=1499)	Females (n=1409)	p-value for sex-difference test
Pubertal status at age 14-15			
fully mature	370 (24.7)		
advanced stage puberty	457 (30.5)		
early stage puberty	512 (34.2)		
prepubescent	160 (10.7)		
Age at menarche in years			
≤ 11		238 (16.9)	
12		402 (28.5)	
13		501 (35.6)	
≥ 14		268 (19.0)	
Leisure-time physical activity in previous month (yes v. no) [a]			
age 36	917 (69.1)	730 (56.9)	*P*<0.001
age 43	671 (51.1)	545 (43.6)	*P*<0.001
age 53	614 (52.2)	573 (48.6)	*P*=0.08
age 60-64	294 (34.2)	335 (37.1)	*P*=0.2
age 68	369 (39.1)	401 (40.7)	*P*=0.5
Birth weight (kg)			*P*<0.001
≤ 2.50 (n=129)	54 (3.6)	75 (5.3)	
2.51-3.00 (n=475)	202 (13.5)	273 (194)	
3.01-3.50 (n=1031)	497 (33.2)	534 (37.9)	
3.51-4.00 (n=982)	547 (36.5)	435 (30.9)	
> 4.00 (n=291)	199 (13.3)	92 (6.5)	
Birth order			*P=0.8*
first born (n=1200)	624 (41.6)	576 (40.9)	
second born (n=953)	482 (32.2)	471 (33.4)	
third or later born (n=755)	393 (26.2)	362 (25.7)	
Serious childhood illness			*P=0.2*
no (n=2723)	1395 (93.1)	1328 (94.3)	
yes (n=185)	104 (6.9)	81 (5.8)	
Father's occupational class age 4			*P=0.9*
professional/managerial/technical (n=650)	341 (n=22.8)	309 (21.9)	
skilled non-manual (n=547)	277 (18.5)	270 (19.2)	
skilled manual (n=902)	459 (30.6)	443 (31.4)	
partly skilled or unskilled (n=809)	422 (28.2)	387 (27.5)	

[a] Proportions are for those taking part (at least once per month) in leisure-time physical activity at each age.

For peer review only - http://bmjopen.bmj.com/site/about/guidelines.xhtml

Table 2 Associations between Pubertal Status at Age 14-15 Years and Leisure-Time Physical Activity (LTPA) at each Adult Age in Men from the MRC NSHD, 1946-2014.

	N (%) LTPA*	Odds ratios (95% confidence intervals) of LTPA at least once per month at each adult age versus no LTPA Model 1	Model 2	Model 3
LTPA age 36 years				
fully mature (n=325)	238 (73.2)	1.00	1.00	1.00
advanced stage puberty (n=403)	263 (65.3)	0.69 (0.50, 0.95)	0.70 (0.51, 0.96)	0.69 (0.50, 0.96)
early stage puberty (n=461)	314 (68.1)	0.78 (0.57, 1.07)	0.79 (0.58, 1.09)	0.78 (0.57, 1.08)
prepubescent (n=139)	102 (73.4)	1.01 (0.64, 1.58)	1.03 (0.65, 1.62)	1.05 (0.67, 1.65)
test of association		*P*=0.08	*P*=0.1	*P*=0.07
LTPA age 43 years				
fully mature (n=322)	182 (56.5)	1.00	1.00	1.00
advanced stage puberty (n=406)	198 (48.8)	0.73 (0.55, 0.98)	0.76 (0.56, 1.02)	0.73 (0.54, 0.99)
early stage puberty (n=445)	224 (50.3)	0.78 (0.58, 1.04)	0.83 (0.62, 1.11)	0.80 (0.60, 1.08)
prepubescent (n=139)	67 (48.2)	0.72 (0.48, 1.07)	0.76 (0.50, 1.14)	0.78 (0.52, 1.18)
test of association		*P*=0.2	*P*=0.3	*P*=0.2
LTPA age 53 years				
fully mature (n=292)	149 (51.0)	1.00	1.00	1.00
advanced stage puberty (n=359)	186 (51.8)	1.03 (0.76, 1.41)	1.07 (0.78, 1.46)	1.06 (0.77, 1.45)
early stage puberty (n=403)	215 (53.4)	1.10 (0.81, 1.48)	1.14 (0.84, 1.54)	1.12 (0.82, 1.53)
prepubescent (n=122)	64 (52.5)	1.06 (0.69, 1.62)	1.10 (0.72, 1.69)	1.16 (0.75, 1.79)
test of association		*P*=0.9	*P*=0.9	*P*=0.9
LTPA age 60-64 years				
fully mature (n=217)	74 (34.1)	1.00 (reference)	1.00 (reference)	1.00 (reference)
advanced stage puberty (n=263)	96 (36.5)	1.11 (0.76, 1.62)	1.13 (0.77, 1.65)	1.12 (0.76, 1.65)
early stage puberty (n=293)	97 (33.1)	0.96 (0.66, 1.40)	0.97 (0.66, 1.41)	0.98 (0.67, 1.43)
prepubescent (n=88)	27 (30.7)	0.86 (0.51, 1.48)	0.90 (0.52, 1.54)	0.94 (0.54, 1.63)
test of association		*P*=0.8	*P*=0.8	*P*=0.9
LTPA age 68 years				
fully mature (n=239)	92 (38.5)	1.00	1.00	1.00
advanced stage puberty (n=296)	116 (39.2)	1.03 (0.73, 1.46)	1.04 (0.73, 1.48)	1.03 (0.72, 1.47)
early stage puberty (n=316)	125 (39.6)	1.05 (0.74, 1.48)	1.03 (0.73, 1.46)	1.04 (0.73, 1.47)
prepubescent (n=93)	36 (38.7)	1.01 (0.62, 1.65)	1.01 (0.62, 1.66)	1.06 (0.65, 1.76)
test of association		*P*>0.9	*P*>0.9	*P*>0.9

Analytic samples consist of those with maximum data at each age. Model 1: unadjusted. Model 2: adjusted for birth weight, birth order and childhood illness. Model 3: as for model 2 plus adjustment for father's occupational class. P-values from test of difference between pubertal groups. *Proportions indicate those taking part in LTPA (at least once per month) at each age.

For peer review only - http://bmjopen.bmj.com/site/about/guidelines.xhtml

Table 3 Associations between Age at Menarche and Leisure-Time Physical Activity (LTPA) at each Adult Age in Women from the MRC NSHD, 1946-2014.

	N (%) LTPA*	Odds ratios (95% confidence intervals) of LTPA at least once per month at each adult age versus no LTPA Model 1	Model 2	Model 3
LTPA age 36 years				
≤ 11 (n=216)	118 (54.6)	1.00	1.00	1.00
12 (n=363)	202 (55.6)	0.96 (0.63, 1.45)	0.96 (0.63, 1.45)	1.19 (0.87, 1.62)
13 (n=464)	276 (59.5)	1.14 (0.76, 1.69)	1.14 (0.76, 1.70)	1.05 (0.74, 1.50)
≥ 14 (n=240)	134 (55.8)	1.06 (0.67, 1.67)	1.07 (0.67, 1.70)	1.68 (0.88, 3.24)
test of association		*P*=0.8	*P*=0.8	*P*=0.8
LTPA age 43 years				
≤ 11 (n=208)	82 (39.4)	1.00	1.00	1.00
12 (n=355)	157 (44.2)	1.16 (0.77, 1.74)	1.17 (0.78, 1.77)	1.27 (0.84, 1.93)
13 (n=440)	191 (43.4)	1.21 (0.82, 1.78)	1.24 (0.84, 1.84)	1.31 (0.88, 1.96)
≥ 14 (n=248)	115 (46.4)	1.24 (0.80, 1.92)	1.29 (0.82, 2.02)	1.35 (0.86, 2.13)
test of association		*P*=0.8	*P*=0.7	*P*=0.5
LTPA age 53 years				
≤ 11 (n=203)	102 (50.3)	1.00	1.00	1.00
12 (n=332)	162 (48.8)	0.91 (0.61, 1.37)	0.91 (0.61, 1.38)	0.99 (0.65, 1.50)
13 (n=406)	189 (46.6)	0.82 (0.56, 1.21)	0.84 (0.57, 1.25)	0.90 (0.60, 1.34)
≥ 14 (n=239)	120 (50.2)	1.03 (0.66, 1.59)	1.05 (0.68, 1.64)	1.10 (0.70, 1.72)
test of association		*P*=0.6	*P*=0.7	*P*=0.8
LTPA age 60-64 years				
≤ 11 (n=148)	52 (35.1)	1.00	1.00	1.00
12 (n=270)	101 (37.4)	1.06 (0.68, 1.66)	1.09 (0.69, 1.72)	1.17 (0.74, 1.86)
13 (n=315)	120 (38.1)	1.24 (0.81, 1.92)	1.31 (0.85, 2.04)	1.39 (0.89, 2.17)
≥ 14 (n=169)	62 (36.7)	1.01 (0.62, 1.66)	1.06 (0.64, 1.75)	1.09 (0.66, 1.81)
test of association		*P*=0.7	*P*=0.6	*P*=0.5
LTPA age 68 years				
≤ 11 (n=162)	66 (40.7)	1.00	1.00	1.00
12 (n=280)	108 (38.6)	0.91 (0.62, 1.36)	0.92 (0.62, 1.38)	1.00 (0.66, 1.50)
13 (n=357)	147 (41.2)	1.02 (0.70, 1.49)	1.01 (0.69, 1.48)	1.08 (0.73, 1.59)
≥ 14 (n=186)	80 (43.0)	1.10 (0.72, 1.68)	1.08 (0.70, 1.66)	1.13 (0.73, 1.76)
test of association		*P*=0.8	*P*=0.9	*P*=0.9

Analytic samples consist of those with maximum data at each age. Model 1: unadjusted. Model 2: adjusted for birth weight, birth order and childhood illness. Model 3: as for model 2 plus adjustment for father's occupational class. P-values from test of difference between ages at menarche groups. *Proportions indicate those taking part in LTPA (at least once per month) at each age.

For peer review only - http://bmjopen.bmj.com/site/about/guidelines.xhtml

Supplementary table 1 Associations between Pubertal Status at Age 15 Years and Leisure-Time Physical Activity (LTPA) at each Adult Age in Men from the MRC NSHD after further adjustment for adult physical health, 1946-2014.

	N (%) LTPA*	Odds ratios (95% confidence intervals) of LTPA at least once per month at each adult age versus no LTPA	
		Model A	Model B
LTPA age 36 years			
fully mature (n=316)	230 (72.8)	1.00	1.00
advanced stage puberty (n=395)	258 (65.3)	0.71 (0.51, 0.98)	0.69 (0.50, 0.96)
early stage puberty (n=455)	308 (67.7)	0.78 (0.57, 1.08)	0.76 (0.55, 1.05)
prepubescent (n=131)	97 (74.1)	1.12 (0.70, 1.78)	1.09 (0.69, 1.75)
test of association		*P*=0.07	*P*=0.06
LTPA age 43 years			
fully mature (n=293)	169 (57.7)	1.00	1.00
advanced stage puberty (n=363)	176 (48.5)	0.69 (0.50, 0.95)	0.68 (0.49, 0.94)
early stage puberty (n=408)	201 (49.3)	0.74 (0.54, 1.00)	0.71 (0.52, 0.98)
prepubescent (n=119)	58 (48.7)	0.78 (0.50, 1.21)	0.75 (0.48, 1.18)
test of association		*P*=0.1	*P*=0.1
LTPA age 53 years			
fully mature (n=258)	135 (52.3)	1.00	1.00
advanced stage puberty (n=314)	164 (52.2)	1.03 (0.73, 1.44)	1.01 (0.72, 1.42)
early stage puberty (n=367)	191 (52.0)	1.03 (0.74, 1.42)	0.98 (0.70, 1.36)
prepubescent (n=106)	54 (51.0)	1.05 (0.66, 1.67)	1.04 (0.65, 1.66)
test of association		*P*>0.9	*P*>0.9
LTPA age 60-64 years			
fully mature (n=195)	66 (33.9)	1.00	1.00
advanced stage puberty (n=235)	85 (36.2)	1.15 (0.76, 1.72)	1.13 (0.75, 1.71)
early stage puberty (n=272)	85 (31.3)	0.92 (0.61, 1.37)	0.87 (0.58, 1.31)
prepubescent (n=82)	23 (28.1)	0.84 (0.47, 1.51)	0.84 (0.46, 1.50)
test of association		*P*=0.6	*P*=0.5
LTPA age 68 years			
fully mature (n=203)	80 (39.4)	1.00	1.00
advanced stage puberty (n=253)	98 (38.7)	0.97 (0.66, 1.42)	0.96 (0.66, 1.42)
early stage puberty (n=289)	114 (39.5)	0.98 (0.68, 1.43)	0.97 (0.67, 1.41)
prepubescent (n=84)	31 (36.9)	0.94 (0.55, 1.60)	0.94 (0.55, 1.60)
test of association		*P*>0.9	*P*>0.9

Analytic samples consist of those with maximum data at each age. Model A: adjusted for birth weight, birth order, childhood illness and father's occupational class. Model B: further adjusted for adult physical health. P-values from test of difference between pubertal groups.

For peer review only

Supplementary table 2 Associations between Age at Menarche and Leisure-Time Physical Activity (LTPA) at each Adult Age in Women from the MRC NSHD after further adjustment for adult physical health, 1946-2014.

		Odds ratios (95% confidence intervals) of LTPA at least once per month at each adult age versus no LTPA	
	N (%) LTPA*	Model A	Model B
LTPA age 36 years			
≤ 11 (n=210)	115 (54.8)	1.00	1.00
12 (n=359)	200 (55.7)	1.07 (0.75, 1.52)	1.03 (0.72, 1.47)
13 (n=457)	270 (59.1)	1.22 (0.87, 1.72)	1.18 (0.84, 1.67)
≥ 14 (n=235)	131 (55.7)	1.08 (0.73, 1.58)	1.00 (0.67, 1.47)
test of association		*P*=0.6	*P*=0.6
LTPA age 43 years			
≤ 11 (n=190)	74 (39.0)	1.00	1.00
12 (n=333)	143 (42.9)	1.25 (0.85, 1.79)	1.22 (0.84, 1.77)
13 (n=407)	173 (42.5)	1.22 (0.85, 1.76)	1.19 (0.83, 1.71)
≥ 14 (n=219)	99 (45.2)	1.36 (0.91, 2.04)	1.30 (0.86, 1.97)
test of association		*P*=0.5	*P*=0.6
LTPA age 53 years			
≤ 11 (n=184)	92 (50.0)	1.00	1.00
12 (n=305)	148 (48.5)	1.01 (0.69, 1.47)	0.99 (0.68, 1.45)
13 (n=377)	170 (45.1)	0.89 (0.61, 1.28)	0.87 (0.60, 1.26)
≥ 14 (n=213)	109 (51.2)	1.13 (0.75, 1.70)	1.08 (0.71, 1.63)
test of association		*P*=0.6	*P*=0.6
LTPA age 60-64 years			
≤ 11 (n=133)	44 (33.1)	1.00	1.00
12 (n=248)	95 (38.3)	1.50 (0.94, 2.39)	1.44 (0.90, 2.30)
13 (n=290)	112 (38.6)	1.56 (0.99, 2.45)	1.54 (0.97, 2.42)
≥ 14 (n=157)	56 (35.7)	1.26 (0.75, 2.09)	1.15 (0.69, 1.93)
test of association		*P*=0.2	*P*=0.2
LTPA age 68 years			
≤ 11 (n=142)	55 (38.7)	1.00	1.00
12 (n=250)	96 (38.4)	1.06 (0.68, 1.63)	1.02 (0.66, 1.57)
13 (n=323)	133 (41.2)	1.15 (0.76, 1.74)	1.13 (0.75, 1.72)
≥ 14 (n=164)	66 (40.2)	1.11 (0.69, 1.78)	1.03 (0.64, 1.66)
test of association		*P*>0.9	*P*=0.9

Analytic samples consist of those with maximum data at each age. Model A: adjusted for birth weight, birth order, childhood illness and father's occupational class. Model B: further adjusted for adult physical health. P-values from test of difference between pubertal groups.

For peer review only

STROBE Guidelines for authors of CORR

To be used by authors of all observational clinical studies published in CORR. For this purpose a cohort study (the term used by STROBE) is considered a longitudinal study typically reporting outcomes of treatment in one or more cohorts; a case-control study is one identifying factors in outcomes; a cross-sectional study is one to identify the prevalence of factors or characteristics in a population at a single point in time.

This table is modified from and used with the permission of The STROBE Initiative, www.strobe-statement.org.

Modifications: We added a fourth column for authors to check inclusion. You must include all items in your manuscript unless the information is not applicable. Information on the study cohort (Items 13 and 14 in the STROBE guidelines) should be provided in Patients and Methods, not in Results; we have omitted the portions of the STROBE guidelines related to Results and Discussion (see our guidelines). The STROBE guidelines were developed for epidemiological studies; "exposed" or "exposure" have been modified with the words "treated" or "treatment."

STROBE Statement—Checklist of items that should be included in reports of ***case-control studies***

	**Item No**	**Recommendation**	**Please insert check where included or N/A where not applicable**
Title and abstract	1	(*a*) Indicate the study's design with a commonly used term in the title or the abstract	1-2
		(*b*) Provide in the abstract an informative and balanced summary of what was done and what was found	2-3
		**Introduction**	
Background/rationale	2	Explain the scientific background and rationale for the investigation being reported	4-5
Objectives	3	State specific objectives, including any prespecified hypotheses	5
		**Methods**	
Study design	4	Present key elements of study design early in the paper	6-8
Setting	5	Describe the setting, locations, and relevant dates, including periods of recruitment, treatment, follow-up, and data collection	6
Participants	6	(*a*) *Cohort study*—Give the eligibility criteria, and the sources and methods of selection of participants. Describe methods of follow-up *Case-control study*—Give the eligibility criteria, and the sources and methods of case ascertainment and control selection. Give the rationale for the choice of cases and controls	6

For peer review only - http://bmjopen.bmj.com/site/about/guidelines.xhtml

Cross-sectional study—Give the eligibility criteria, and the sources and methods of selection of participants	
(*b*) *Cohort study*—For matched studies, give matching criteria and number of treated and untreated<br>*Case-control study*—For matched studies, give matching criteria and the number of controls per case	NA

For peer review only

For peer review only - http://bmjopen.bmj.com/site/about/guidelines.xhtml

Participants	13*	(a) Report numbers of individuals at each stage of study—eg numbers potentially eligible, examined for eligibility, confirmed eligible, included in the study, completing follow-up, and analysed	6
		(b) Give reasons for non-participation at each stage	6-7
Descriptive data	14*	(a) Give characteristics of study participants (eg demographic, clinical, social) and information on other treatments and potential confounders	10, table 1
		(b) Indicate number of participants with missing data for each variable of interest	6-7, 10
		(c) *Cohort study*—Summarise follow-up time (eg, average and total amount)	6-7
Variables	7	Clearly define all outcomes, treatments, predictors, potential confounders, and effect modifiers. Give diagnostic criteria, if applicable	7-9
Data sources/ measurement	8*	For each variable of interest, give sources of data and details of methods of assessment (measurement). Describe comparability of assessment methods if there is more than one group	6-9
Bias	9	Describe any efforts to address potential sources of bias	7-9, 13-14
Study size	10	Explain how the study size was arrived at	6-7
Quantitative variables	11	Explain how quantitative variables were handled in the analyses. If applicable, describe which groupings were chosen and why	7-8
Statistical methods	12	(*a*) Describe all statistical methods, including those used to control for confounding	8-9
		(*b*) Describe any methods used to examine subgroups and interactions	8-9
		(*c*) Explain how missing data were addressed	7-10
		(*d*) *Cohort study*—If applicable, explain how loss to follow-up was addressed<br>*Case-control study*—If applicable, explain how matching of cases and controls was addressed<br>*Cross-sectional study*—If applicable, describe analytical methods taking account of sampling strategy	6-10
		(*e*) Describe any sensitivity analyses	7-9, 14

*Give information separately for cases and controls.

Note: An Explanation and Elaboration article discusses each checklist item and gives methodological background and published examples of transparent reporting. The STROBE checklist is best used in conjunction with this article (freely available on the Web sites of PLoS Medicine at http://www.plosmedicine.org/, Annals of Internal Medicine at http://www.annals.org/, and Epidemiology at http://www.epidem.com/). Information on the STROBE Initiative is available at http://www.strobe-statement.org.

For peer review only - http://bmjopen.bmj.com/site/about/guidelines.xhtml
